# Effect of Adding a Work-Focused Intervention to Integrated Care for Depression in the Veterans Health Administration: A Randomized Clinical Trial

**DOI:** 10.1001/jamanetworkopen.2020.0075

**Published:** 2020-02-28

**Authors:** Debra Lerner, David A. Adler, William H. Rogers, Erin Ingram, David W. Oslin

**Affiliations:** 1 Program on Health, Work and Productivity, Institute for Clinical Research and Health Policy Studies, Tufts Medical Center, and Tufts University School of Medicine and Tufts Graduate School of Biomedical Sciences, Boston, Massachusetts; 2 Department of Psychiatry, Tufts Medical Center, Boston, Massachusetts; 3 Veterans Integrated Service Network 4 Mental Illness Research, Education, and Clinical Center, Center of Excellence, Corporal Michael J. Crescenz VA Medical Center, Philadelphia, Pennsylvania; 4 Department of Psychiatry, Perelman School of Medicine, University of Pennsylvania, Philadelphia

## Abstract

**Importance:**

Thousands of working-age veterans with depression experience impaired occupational functioning.

**Objectives:**

To test whether the Veterans Health Administration (VHA) integrated care (IC) program combined with telephonic work-focused counseling, known as *Be Well at Work* (BWAW), is superior to IC alone for improving occupational functioning and depression, to determine whether these effects persist 4 months later, and to determine whether the return on investment is positive.

**Design, Setting, and Participants:**

In this randomized clinical trial conducted from October 21, 2014, to December 6, 2019, patients undergoing IC at VHA facilities were screened for eligibility and randomized to IC alone or IC plus BWAW. Blinded interviewers administered questionnaires before the intervention, immediately after completion of the intervention at month 4, and at month 8. Eligibility criteria were individuals 18 years or older who were working at least 15 hours per week in a job they had occupied for at least 6 months, were experiencing work limitations, and had current major depressive disorder or persistent depressive disorder. Exclusion criteria were individuals who could not read or speak English, had planned maternity leave, or had a history of bipolar disorder or psychosis. Data analyses were conducted from January 1, 2018, to December 6, 2019.

**Interventions:**

Integrated care is multidisciplinary depression care involving screening, clinical informatics, measurement-based care, brief behavioral interventions, and referral as needed to specialty mental health care. Be Well at Work counseling involves 8 biweekly telephone sessions and 1 telephone booster visit after 4 months. Doctoral-level psychologists helped patients to identify barriers to functioning and to adopt new work-focused cognitive-behavioral and work-modification strategies.

**Main Outcomes and Measures:**

The primary outcome was the adjusted mean group difference in changes from before to after intervention (hereafter, *adjusted effect*) in the percentage of at-work productivity loss, measured with the Work Limitations Questionnaire (range, 0%-25%). The secondary outcome was adjusted effect in the Patient Health Questionnaire 9-item symptom severity score (range, 0-27, with 0 indicating no symptoms and 27, severe symptoms).

**Results:**

Of 670 veterans referred for participation, 287 veterans (42.8%) consented and completed eligibility screening, and 253 veterans (37.8%) were randomized. Among these 253 patients (mean [SD] age, 45.7 [11.6] years; 218 [86.2%] men; 135 [53.4%] white), 114 (45.1%) were randomized to IC and 139 (54.9%) were randomized to IC plus BWAW. At the 4-month follow-up, patients who received IC plus BWAW had greater reductions in at-work productivity loss (adjusted effect, −1.7; 95% CI, −3.1 to −0.4; *P* = .01) and depression symptom severity (adjusted effect, −2.1; 95% CI, −3.5 to −0.7; *P* = .003). The improvements from IC plus BWAW persisted 4 months after intervention (at-work productivity loss mean difference, −0.5; 95% CI, −1.9 to 0.9; *P* = .46; depression symptom severity mean difference, 0.6; 95% CI −0.9 to 2.1; *P* = .44). The cost per patient participating in BWAW was $690.98, and the return on investment was 160%.

**Conclusions and Relevance:**

These findings suggest that adding this work-focused intervention to IC improves veterans’ occupational and psychiatric outcomes, reducing obstacles to having a productive civilian life.

**Trial Registration:**

ClinicalTrials.gov Identifier: NCT02111811

## Key Points

**Question:**

Is a telephonic work-focused counselling program combined with the Veterans Health Administration’s mental health integrated care (IC) program superior to IC alone for improving the occupational functioning and depression symptom severity of employed veterans with depression and work limitations?

**Findings:**

In this randomized clinical trial including 253 veterans, IC plus work-focused counseling resulted in significantly larger reductions in at-work productivity loss and depression symptom severity immediately after the intervention, and these effects were present 4 months later.

**Meaning:**

These findings suggest that supplementing the IC program with this low-cost, work-focused intervention may help veterans with depression and work limitations achieve better occupational and clinical outcomes.

## Teaser

This randomized clinical trial examines the effect of telephonic work-focused counselling combined with integrated care vs integrated care alone among veterans with depression treated at a large Veterans Health Administration medical center.

## Introduction

In the largest health care system in the United States, the Veterans Health Administration (VHA), approximately 7% of patients meet criteria for major depressive disorder, and 13.5% of patients visiting VHA primary care clinics have depression symptoms.^1^ Major depressive disorder is a costly, frequently disabling condition.^2^ Depression specifically contributes to occupational disability.^3^ Compared with veterans without depression, veterans with depression have more unemployment,^3^ and those who work have more functional deficits (known as *presenteeism*) and work absences.^4,5^ A 2011 study^4^ among Operation Enduring Freedom and Operation Iraqi Freedom veterans who were referred by VHA primary care clinicians for psychiatric evaluation found that veterans with depression had a mean of approximately 6% to 8% less productivity than a healthy benchmark sample.

The VHA currently offers vocational rehabilitation services, which are primarily for unemployed veterans, as well as comprehensive depression care. Since 2007, the VHA has implemented evidence-based, primary care mental health integrated care (IC) for depression, which consists of screening, clinical informatics, measurement-based care, brief behavioral interventions, and referral to specialty mental health care to reduce symptom severity, delivered by multidisciplinary teams.^6^ A 2006 study^7^ of the IC program found that it improved depression detection and treatment as well as clinical outcomes. However, while studies conducted in non-VHA settings report improved occupational outcomes associated with IC,^8,9^ to our knowledge, the effect of IC on the occupational outcomes of veterans with depression is not known.

This study’s primary aim was to test whether the VHA’s primary care mental health IC program for depression in combination with a telephone-based work-focused intervention, known as *Be Well at Work* (BWAW), was more effective than IC alone. In 2 prior clinical trials involving commercially insured employees principally recruited through their employers, BWAW was superior to usual depression care for reducing presenteeism, absenteeism, and depression symptom severity.^10,11^ To our knowledge, this is the first study to test this program’s effectiveness in the context of a health care delivery system in which standard depression care is high quality and evidence based. We hypothesized that, compared with IC alone, the combined program would result in greater improvements in occupational functioning and depression symptom severity and that improvements would persist. We also aimed to determine whether the experimental program provided a return on investment (ROI).

## Methods

From October 21, 2014, through December 6, 2019, we conducted a 2-group randomized clinical trial comparing IC alone with IC combined with BWAW at a large VHA medical center and 2 smaller satellite sites. In this single-blind study, research assistants collecting study data were blinded to treatment group assignment. The study did not restrict veterans’ use of VHA or non-VHA care. Full details on the study protocol are available in Supplement 1, and information on deviations from the protocol are listed in the eAppendix in Supplement 2. The protocol was reviewed and approved by each local institutional review board. All participants provided written informed consent. This study is reported following the Consolidated Standards of Reporting Trials (CONSORT) reporting guideline.

Eligible veterans were 18 years or older, worked at least 15 hours per week in jobs they had occupied for at least 6 months, and had work limitations resulting in at least 5% at-work productivity loss based on validated questionnaire assessment, as well as current major depressive disorder or persistent depressive disorder confirmed by diagnostic interview. Exclusions were inability to speak or read English, planned maternity leave, or a history of bipolar disorder or psychosis.

The IC program referred veterans to the study. Research assistants conducted the pretrial screening, involving a health record review and telephone interview. Potentially eligible, interested patients were invited to come in for informed consent and to complete a further eligibility assessment, which also served as the study baseline measurement (T_0_). For this initial assessment, the research assistant administered standardized questions to assess depression (Patient Health Questionnaire [PHQ-9]; range, 0-27, with 0 indicating no symptoms and 27, severe symptoms),^12^ work limitations (Work Limitations Questionnaire [WLQ]; range, 0%-25%),^13^ mean number of alcoholic drinks per week,^14^ generalized anxiety disorder (General Anxiety Disorder 7-item questionnaire; range, 0-21, in which 0 = no symptoms to 21 = severe anxiety),^15^ posttraumatic stress disorder based on a checklist (1 = present, 0 = absent),^16^ use of illicit substances (excluding cannabis) more than weekly in the past month,^17^ suicidal ideation,^12^ and the Veterans Rand 12-item health survey.^18^

Next, VHA study clinicians administered the Structured Clinical Interview for *Diagnostic and Statistical Manual of Mental Disorders* (Fifth Edition) (DSM-5)^19^ (SCID-5) modules to diagnose major depressive disorder and persistent depressive disorder and rule out bipolar disorder and psychosis.^20^ Finally, patients were asked to complete a self-report questionnaire measuring work absences (WLQ Time Loss Module), work hours, job earnings, demographic characteristics needed for descriptive purposes (eg, race/ethnicity and sex), use of non-VA care, and current and past service-connected disability claims. Physical and psychosocial job demands, job autonomy, and workplace social support were measured with a modified Job Content Questionnaire (range, 0-100, in which 0 indicates minimum and 100, maximum).^21^ These assessments required approximately 1 hour to complete.

If all eligibility criteria were met, the patient was assigned by simple randomization to 1 of the treatment groups. In this automated process, a random uniform number was calculated using the *Math.random* function in Java Math Library random function software version 8 (Oracle Technology Network) and the patient was assigned to IC plus BWAW half of the time.

Follow-up telephone interviews of randomized patients were conducted after the intervention period at month 4 (T_1_), and at approximately months 8 to 9 (T_2_). Patients who did not complete the T_1_ follow-up remained eligible for T_2_. For difficult-to-reach patients, multiple telephone and/or mail outreach attempts were made. At T_2_, we extracted VHA data for 2013 to 2017 on all diagnoses, health care use, and mental health or behavioral health treatments. Incentives included $25 for the baseline visit, $25 for the T_1_ follow-up, and $45 for the T_2_ follow-up (total, $95).

### Interventions

Within IC, patients with mild to moderate disorders were treated in primary care in collaboration with the primary care clinician and an IC mental health care practitioner. Patients with more severe symptoms were encouraged to engage in the appropriate VHA specialty care (eg, mental health services, addiction counseling).^22^ Practitioners in the IC team included psychologists, nurses, and social workers, supervised by a VHA psychiatrist, who supported the use of brief evidence-based psychotherapy and pharmacotherapy. Treatment plans promoted adherence to prescribed antidepressants as well as activities to increase positive social interactions, healthy living, and self-esteem. Patients were continuously assessed to determine whether a higher level of care was needed and to facilitate transition as needed to more specialized services.

Patients assigned to IC plus BWAW also received an individualized program of eight 50-minute telephone visits occurring biweekly for 4 months, and 1 booster session approximately 4 months later. Telephone care reduces the burden of requiring in-person visits for patients who also work. Two doctoral-level psychologists who were not providing IC provided BWAW counseling under the supervision of its developers (a psychiatrist [D.A.A.] and a workplace health specialist [D.L.]). Initially, counselors received an intensive 2.5-day training session, followed by weekly telephone supervision involving in-depth case reviews. Additionally, counselor fidelity to the protocol was monitored through review of electronic health records. Patient records had mandatory data fields to document steps in the BWAW care process (eg, homework assignments, referrals to specialty care, standardized assessments, physician reporting, self-care planning). We did not audiorecord patient care visits.

The BWAW intervention is based on stress and coping theory in which successful mental health and functional outcomes are related to responding effectively to psychosocial challenges.^23^ Accordingly, stress occurs when coping methods are ineffective for confronting life events and experiences, such as work demands, which the person perceives as threatening or harmful. Coping may be compromised by factors such as depression symptoms, access to social supports, and adverse life experiences. Be Well at Work targets 3 areas of coping. First, counselors address issues related to coping with depression treatment using motivational enhancement and psychoeducational strategies to prepare the patient to actively engage in treatment provided in BWAW and by the person’s regular health care practitioner. To coordinate BWAW with existing depression care, counselors assess occupational functioning and depression symptom severity monthly, sharing results with health care practitioners through the VHA electronic health record. Second, counselors provide work-focused cognitive-behavioral therapy strategy training based on a self-help manual adapted from Simon and colleagues.^24^ This component is aimed at increasing the patient’s ability to identify maladaptive patterns of thinking, feeling, and behaving that affect work and substitute more adaptive coping behaviors. Third, the patient and counselor identify and address workplace barriers to effective functioning and potential work-appropriate coping strategies. Drawing from psychology, occupational health, and disability and rehabilitation practices,^25,26^ patients are guided to make small specific changes in how they perform work tasks (eg, new time management techniques), work routines (eg, collaborating vs staying isolated), and/or the work environment (eg, rearranging the workspace). Some patients may be guided to develop compensatory work strategies (eg, using memory aids).

With 2-week intervals between sessions, patients are assigned homework to test new strategies and integrate their use. The BWAW intervention culminates with the development of a customized self-care plan. At the booster session, self-care progress is reviewed, and if necessary, the plan is adjusted.

### Hypotheses and Measures

We hypothesized that T_1_ outcomes of the IC plus BWAW group would be better than those of the IC-only group, indicated by the adjusted mean group difference in the changes in WLQ at-work productivity loss score from before the intervention to after. Secondary end points included the adjusted mean group difference in changes in depressive symptom severity and in productivity loss due to work absences from before the intervention to after. We also assessed adjusted mean differences in the change in percentage of job loss (for any reason) and new disability claims, and in mean weekly work hours. Our second hypothesis was that the improvements observed at T_1_ would be present at T_2_. Our third hypothesis was that the ROI for IC plus BWAW would be positive.

The WLQ at-work productivity loss score is computed using an empirically based algorithm reflecting the observed association between its 4 scale scores and objectively measured productivity. The WLQ assesses the amount of health-related difficulty in the past 2 weeks performing work tasks.^13^ The tasks are organized into work limitation scales addressing time management, physical tasks, mental and interpersonal tasks, and output tasks. Scale scores range from 0% (limited none of the time) to 100% (limited all of the time). Scale Cronbach α in this sample are 0.87 to 0.89. The at-work productivity loss score is the weighted sum of the scale scores, and quantifies the estimated percentage difference in at-work productive output between a person (or group) completing the WLQ and an external healthy worker benchmark sample (range, 0%-25%, with a higher score indicating more at-work productivity loss).^27^ The WLQ Time Loss Module measures the number of hours of work missed in the past 2 weeks owing to a health-related issue. The percentage of productivity loss due to absences was calculated as the number of hours missed divided by the number of hours usually spent working. Depression symptom severity was measured with the Patient Health Questionnaire, with scores ranging from 0, indicating no depressive symptoms to 27, severe depression.^28^ The sample Cronbach α is 0.78.

### Statistical Analysis

For our first hypothesis, based on data from the primary site we estimated that a sample size of 100 enrollees per treatment group would be needed for 80% power to detect a mean group difference in the change in at-work productivity loss score of 1.2% at T_1_, based on an intent-to-treat analysis using analysis of covariance (ANCOVA) with a 2-sided *t* test and statistical significance set at *P* = .05. For our second hypothesis, we used mixed models testing for maintenance of outcomes from T_1_ to T_2_.

From an economic perspective, a 1–percentage point productivity improvement is meaningful at group, company, and national levels. For example, a 2% to 3% change in output nationally is regarded as an important indicator.^29^ On an employer level, if 6% of employees in a 10 000-person company with mean annual earnings of $45 000 were to have depression, a 1% improvement in productivity would generate productivity savings of $450 per employee with depression, or $270 000 companywide.

Prior to testing our hypothesis, we evaluated the randomization with *t* tests and χ^2^ statistics, the effect of attrition from the study, and BWAW treatment drop-out. Because the 2 low-volume satellite sites enrolled only 3 patients, their data were excluded.

For our first hypothesis, we present the mean change in the primary study outcome by group followed by hypothesis-testing of the adjusted T_1_ outcome with ANCOVA (adjusted mean group difference in changes from before to after intervention [hereafter, *adjusted effect*] and *P* value). All other study outcomes (ie, depression symptom severity, productivity loss due to absences, job loss, disability claims, and weekly work hours) were tested using these methods. Additionally, mixed-model results based on all available data and a last value–carry forward analysis were used to confirm that sample losses to follow-up did not bias conclusions.

For our second hypothesis, changes from T_1_ to T_2_ and intervention effects were estimated and tested using mixed models. Models included variables for time and a treatment-by-time interaction. The T_2_ follow-up interaction was compared with the T_1_ interaction using 2-sided *z* tests with *P* = .05.

For our third hypothesis evaluating ROI, we computed the ratio of benefits minus costs to costs.^30^ To monetize benefits, we computed the marginal benefits at T_2_ in at-work productivity loss and productivity loss due to absences using a mean of 4- and 8-month effects, and combined them with the formula: *total productivity los*s = *absenteeism loss* + *at-work productivity loss* × (*1* – *absenteeism*). Next, total percentage loss was multiplied by the sample’s median annual job earnings, $45 000. Marginal costs were based on the mean number of counselor hours per BWAW participant. Counselor cost per clinical hour was calculated as the sum of a doctoral-level counselor salary ($105 697) plus benefits (32% of salary) and overhead (20% of salary), divided by 1260 clinical counseling hours available per year, totaling $132.88 per hour. The total cost of participating in BWAW was computed as this hourly amount multiplied by the number of per-participant treatment hours (mean [SD], 5.2 [2.9] hours). Missed appointment hours and supervision time were considered part of counselor time other than the included 1260 clinical counseling hours.

All analyses were performed with Stata statistical software version 15 (StataCorp). Data analyses were conducted from January 1, 2018, to December 6, 2019.

## Results

Of 670 veterans referred to the study, 143 (21.3%) were ineligible at pretrial screening, 142 (21.2%) could not be reached for eligibility screening, and 98 (14.6%) declined further assessment (Figure). Of 287 veterans (42.8%) who consented to the study, 31 (4.6%) were ineligible at T_0_ assessment, and 3 (0.4%) dropped out. Therefore, the starting sample included 253 patients (37.8% of those originally referred; mean [SD] age, 45.7 [11.6] years; 218 [86.2%] men) (Table 1). There were 135 white patients (53.4%), and 116 patients (46.2%) reported having attended some college. At T_0_, 114 patients (45.1%) were randomized to IC and 139 patients (54.9%) were randomized to IC plus BWAW. The T_1_ follow-up survey was completed by 96 patients (83.5%) in the IC group and 115 patients (82.7%) in the IC plus BWAW group, and the T_2_ follow-up survey was completed by 97 patients (84.3%) in the IC group and 111 patients (79.9%) in the IC plus BWAW group (study attrition rate, 17.8%). At baseline (Table 1 and Table 2) and after attrition (eTable 1 and eTable 2 in Supplement 2), the treatment groups were not significantly different on any variable.

**Figure.**
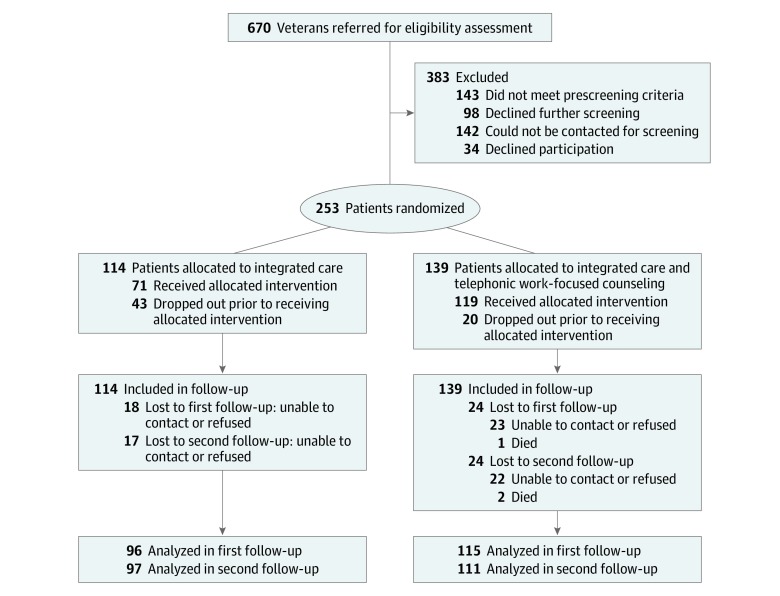
CONSORT Flow Diagram

**Table 1.** Baseline Sample Characteristics of IC-Only Group vs IC Plus BWAW Group

Characteristic	No. (%)				*P* Value for Difference
	Total Respondents	Total (N = 253)	IC Only (n = 114)	IC + BWAW (n = 139)	
Age, mean (SD), y	253	45.7 (11.6)	44.8 (11.6)	46.5 (11.6)	.26
Men	253	218 (86.2)	96 (84.2)	122 (87.8)	.41
White race	253	135 (53.4)	56 (49.1)	79 (56.8)	.22
Education					
<High school	351	2 (0.8)	2 (1.8)	0	.50
High school	251	44 (17.5)	18 (15.8)	26 (19.0)	
Some college	251	116 (46.2)	57 (50.0)	59 (43.1)	
Associate’s degree	251	19 (7.6)	7 (6.1)	12 (8.8)	
Bachelor’s degree	251	40 (15.9)	18 (15.8)	22 (16.1)	
Postgraduate degree	251	30 (12.0)	12 (10.5)	18 (13.1)	
Annual earnings, median, (range), $	243	45 000 (500-200 000)	45 000 (500-140 000)	45 000 (500-200 000)	.99
Work status					
Working for pay	251	237 (94.4)	104 (91.2)	133 (97.1)	.11
Off work owing to illness	251	4 (1.6)	4 (3.5)	0	
Off work owing to injury	251	2 (0.8)	1 (0.9)	1 (0.7)	
On vacation	251	6 (2.4)	3 (2.6)	3 (2.2)	
Not working (other reason)	251	2 (0.8)	2 (1.8)	0	
Time at current job, y					
<1	251	52 (20.7)	22 (19.3)	30 (21.9)	.53
1 to <2	251	48 (19.1)	26 (22.8)	22 (16.1)	
2 to <5	251	46 (18.3)	19 (16.7)	27 (19.7)	
5 to 10	251	44 (17.5)	17 (14.9)	27 (19.7)	
>10	251	61 (24.3)	30 (26.3)	31 (22.6)	
Job type					
Permanent	251	223 (88.8)	102 (89.5)	121 (88.3)	.77
Temporary	251	28 (11.2)	12 (10.5)	16 (11.7)	
Payment structure					
Hourly	250	158 (63.2)	70 (61.4)	88 (64.7)	.59
Salaried	250	87 (34.8)	42 (36.8)	45 (33.1)	.54
Member of labor union	251	101 (40.2)	43 (37.7)	58 (42.3)	.46
Employment type					
Self-employed	249	12 (4.8)	4 (3.6)	8 (5.8)	.41
Private company	249	117 (47.0)	59 (52.7)	58 (42.3)	.10
Government agency	249	120 (48.2)	49 (43.8)	71 (51.8)	.21
Filed disability claim, past 4 mo	251	10 (4.0)	7 (6.1)	3 (2.2)	.11
Insurance through employer	251	137 (54.6)	61 (53.5)	76 (55.5)	.76
Eligible for paid sick time	249	187 (75.1)	84 (75.0)	103 (75.2)	.97
PHQ-9 symptom severity score, mean (SD)		14.5 (4.8)	14.1 (4.9)	14.8 (4.8)	.25
High suicide risk	253	54 (21.3)	25 (21.9)	29 (20.9)	.84
SCID results					
MDD and PDD	253	107 (42.3)	45 (39.5)	62 (44.6)	.41
MDD	253	106 (41.9)	50 (43.9)	56 (40.3)	.57
PDD	253	40 (15.8)	19 (16.7)	21 (15.1)	.74
GAD-7 score, mean (SD)	252	13.0 (4.8)	12.8 (4.5)	13.1 (5.0)	.62
PTSD	253	151 (59.7)	69 (60.5)	82 (59.0)	.81
Alcoholic drinks/wk, mean (SD), No.	251	5.4 (8.9)	5.1 (8.8)	5.6 (8.9)	.64
Recent substance misuse	253	5 (2.0)	3 (2.6)	2 (1.4)	.50
VR12 score, mean (SD)					
Physical component	251	38.3 (12.9)	38.2 (13.4)	38.4 (2.4)	.94
Mental component	251	28.5 (10.8)	29.0 (11.1)	28.1 (10.6)	.49
Using antidepressant medication	253	68 (26.9)	26 (22.8)	42 (30.2)	.19
Non-VHA health care, past 4 mo					
Physician visit	251	95 (37.8)	44 (38.6)	51 (37.2)	.82
Therapist visit	251	23 (9.2)	13 (11.4)	10 (7.3)	.26
Hospitalization	251	7 (2.8)	4 (3.5)	3 (2.2)	.53
Emergency department visit	251	28 (11.2)	13 (11.4)	15 (10.9)	.91

Abbreviations: BWAW, Be Well at Work; GAD-7, 7-item General Anxiety Disorder questionnaire; IC, integrated care; MDD, major depressive disorder; PDD, persistent depressive disorder; PHQ-9, 9-item Patient Health Questionnaire; PTSD, posttraumatic stress disorder; SCID, Structured Clinical Interview for *Diagnostic and Statistical Manual of Mental Disorders* (Fifth Edition); VHA, Veterans Health Administration; VR12, Veterans Rand 12-item health survey.

**Table 2.** Baseline Self-Reported Work Issues Among IC-Only vs IC Plus BWAW Groups

Self-Reported Work Issue	Respondents, No.	Mean (SD)			*P* Value for Difference
		Total	IC Only	IC + BWAW	
Work Limitations Questionnaire score^a^					
Time management	250	52.0 (23.5)	51.3 (24.5)	52.6 (22.7)	.65
Physical tasks	248	41.3 (24.7)	41.8 (24.1)	40.9 (25.2)	.78
Mental or interpersonal tasks	251	46.8 (21.4)	46.9 (21.3)	46.8 (21.5)	.96
Output tasks	249	46.1 (24.3)	46.4 (23.9)	45.9 (24.8)	.89
At-work productivity loss, %^b^	245	12.3 (4.7)	12.3 (4.5)	12.4 (4.9)	.88
Absenteeism					
Time missed in the past 2 wk, d	253	1.8 (2.4)	1.9 (2.4)	1.8 (2.3)	.55
Productivity loss due to absences, %^c^	250	18.6 (22.4)	20.7 (25.0)	16.9 (20.1)	.19
Had leave of absence in past 4 mo, No. (%)	249	32 (12.9)	16 (14.3)	16 (11.7)	.54
Modified Job Content Questionnaire scores					
Job control	251	47.5 (21.2)	48.0 (21.2)	47.1 (21.4)	.74
Physical job demands	251	46.7 (31.5)	44.2 (30.9)	48.8 (32.0)	.25
Psychosocial job demands	251	61.7 (18.5)	61.6 (18.2)	61.8 (18.8)	.93
Supervisor support	251	52.8 (32.2)	54.4 (31.9)	51.5 (32.5)	.47
Coworker support	250	57.6 (29.4)	58.8 (28.2)	56.6 (30.5)	.57
Bothered or upset by work, No. (%)					
Almost always	251	43 (17.1)	17 (14.9)	26 (19.0)	.18
Often	251	102 (40.6)	47 (41.2)	55 (40.1)	
Sometimes	251	88 (35.1)	45 (39.5)	43 (31.4)	
Rarely	251	17 (6.8)	4 (3.5)	13 (9.5)	
Never	251	1 (0.4)	1 (0.9)	0 (0)	
Job satisfaction, No. (%)					
Extremely satisfied	251	14 (5.6)	5 (4.4)	9 (6.6)	.81
Very satisfied	251	50 (19.9)	22 (19.3)	28 (20.4)	
Somewhat satisfied	251	107 (42.6)	53 (46.5)	54 (39.4)	
Somewhat dissatisfied	251	55 (21.9)	23 (20.2)	32 (23.4)	
Very dissatisfied	251	25 (10.0)	11 (9.6)	14 (10.2)	

Abbreviations: BWAW, Be Well at Work; IC, integrated care.

^a^ Scores indicate the mean percentage of time in the prior 2 weeks the person was limited by health conditions.

^b^ Range, 0% to 25%.

^c^ Measured as the mean percentage of total hours missed from work in the past 2 weeks owing to health conditions or medical care divided by the number of hours usually spent working (range, 0-100).

Regarding work characteristics, 52 patients (20.7%) had their jobs less than 1 year, while job tenure exceeded 10 years for 61 patients (24.3%); 223 patients (88.8%) were permanent workers; 101 patients (40.2%) had unionized positions; and 158 patients (63.2%) were paid hourly. Most patients had access to employer-sponsored health insurance (137 patients [54.6%]) and paid sick days (187 patients [75.1%]) (Table 1). Similar proportions of patients worked in the private (117 patients [47.0%]) and public (120 patients [48.2%]) sectors. In the prior 4 months, 10 patients (4.0%) had filed for service-connected disability benefits.

At T_0_, the mean (SD) Patient Health Questionnaire depression symptom severity score was 14.5 (4.8), indicating moderate severity; 106 patients (41.9%) met DSM-5 criteria^19^ for major depressive disorder only, 40 patients (15.8%) met criteria for persistent depressive disorder only, and 107 patients (42.3%) met criteria for both. A total of 68 patients (26.9%) were using antidepressant medications at baseline. Overall mean (SD) Veterans Rand 12-item health survey scores for physical health (38.3 [12.9]) and mental health (28.5 [10.8]) components were poor (Table 1).

In the 2 weeks prior to baseline, the mean (SD) proportions of time patients had work limitations were 52.0% (23.5%) for time management, 41.3% (24.7%) for physical tasks, 46.8% (21.4%) for mental and interpersonal tasks, and 46.1% (24.3%) for output tasks (Table 2). Mean (SD) at-work productivity loss was 12.3% (4.7%). In this same period, patients missed a mean (SD) of 1.8 (2.4) workdays and had a mean (SD) absence productivity loss of 18.6% (22.4). There were no significant differences between the IC and IC plus BWAW groups in their ratings of job stress, job satisfaction, job control, job demands, or workplace support. The results from the Modified Job Content Questionnaire suggest that jobs were viewed as more demanding psychosocially (mean [SD] score, 61.7 [18.5]) than physically (mean [SD] score, 46.7 [31.5]).

During the 4-month intervention period, patients in the IC group had a mean (SD) of 3.9 (3.9) mental health office visits compared with 3.8 (5.4) mental health visits in the IC plus BWAW group (*P* = .20). The mean (SD) number of BWAW sessions attended was 5.2 (2.9). Twenty patients (14.2%) in the IC plus BWAW group attended no BWAW sessions or only 1. Approximately one-fifth of patients in the IC plus BWAW group (27 patients [22.1%]) completed the BWAW booster session.

For our first hypothesis, tested with ANCOVA, we found that IC plus BWAW resulted in larger reductions in at-work productivity loss by T_1_ (adjusted effect, −1.7; 95% CI, −3.1 to −0.4; *P* = .01) (Table 3). Regarding secondary outcomes, we found significantly greater reductions in depression symptom severity for the IC plus BWAW group compared with the IC-only group (adjusted effect, −2.1; 95% CI, −3.5 to −0.7; *P* = .003), although both groups experienced significant improvements (IC-only: mean change, −1.6; 95% CI, −2.6 to −0.7; *P* = .001; IC plus BWAW: mean change, −3.8; 95% CI, −4.8 to −2.7; *P* < .001). Although rates of productivity loss due to work absences showed a greater change in the IC plus BWAW group (mean change, −4.0%; 95% CI, −8.5% to 0.5%) than the IC-only group (mean change, −1.3%; 95% CI, −6.9% to 4.2%), the difference was not statistically significant (*P* = .20), but the mean changes from both follow-ups combined was significant (combined effect: at-work productivity loss, –1.94; 95% CI, –0.82 to –3.1; *P* < .001; productivity loss due to absences, 1.96; 95% CI, –7.5 to 3.6; *P* < .001). In terms of additional outcomes assessed, we found that the IC-only group had a significantly greater loss in the number of weekly work hours than the IC plus BWAW group (adjusted effect, 6.4; 95% CI, 2.8 to 10.1; *P* = .001). Changes in the percentages of job loss (adjusted effect, −0.02; 95% CI, −0.08 to 0.03; *P* = .46) and disability claims (adjusted effect, −0.7; 95% CI, −6.3 to 4.9; *P* = .81) were not statistically different. These results were consistent in models based on last value carried–forward analysis (eTable 4 in Supplement 2).

**Table 3.** Analysis of Covariance Among IC-Only vs IC Plus BWAW Groups

Outcome	IC Only				IC + BWAW				Adjusted Effect (95% CI)^a^	*P* Value
	Mean (SD)		Change, Mean (95% CI)	*P* Value	Mean (SD)		Change, Mean (95% CI)	*P* Value		
	T_0_ (n = 96)	T_1_ (n = 96)			T_0_ (n = 115)	T_1_ (n = 115)				
At-work productivity loss, %^b^	11.7 (4.7)	11.7 (4.6)	−0.1 (−1.1 to 0.9)	.86	12.3 (4.9)	10.2 (6.0)	−2.1 (−3.1 to −1.0)	<.001	−1.7 (−3.1 to −0.4)	.01
Depression symptom severity score^c^	14.3 (4.8)	12.6 (5.5)	−1.6 (−2.6 to −0.7)	.001	14.4 (5.3)	10.6 (6.3)	−3.8 (−4.8 to −2.7)	<.001	−2.1 (−3.5 to −0.7)	.003
Productivity loss due to absence, %^d^	19.2 (23.3)	17.9 (21.1)	−1.3 (−6.9 to 4.2)	.64	17.7 (20.1)	13.7 (20.0)	−4.0 (−8.5 to 0.5)	.09	−3.7 (−9.3 to 1.9)	.20
Had leave of absence, %	12.0 (32.6)	9.8 (29.9)	−2.2 (−8.2 to 3.9)	.48	12.6 (33.3)	9.0 (28.8)	−3.6 (−10.7 to 3.5)	.32	−1.0 (−8.5 to 6.4)	.79
Filed disability claim, %	7.4 (26.4)	5.3 (22.6)	−2.1 (−8.0 to 3.8)	.48	1.8 (13.4)	3.6 (18.7)	1.8 (−2.5 to 6.1)	.42	−0.7 (−6.3 to 4.9)	.81
Experienced job loss, %	0	3.1 (17.5)	3.1 (−0.4 to 6.6)	.08	0	5.2 (22.3)	5.2 (1.1 to 9.3)	.01	2.1 (−3.4 to 7.6)	.46
Work hr/wk	45.0 (19.6)	36.6 (13.0)	−8.4 (−12.8 to −4.0)	<.001	43.1 (15.9)	42.6 (14.2)	−0.5 (−3.5 to 2.5)	.73	6.4 (2.8 to 10.1)	.001

Abbreviations: BWAW, Be Well at Work; IC, integrated care; T_0_, baseline; T_1_, 4-month follow-up.

^a^ Controlled for baseline values of the outcome variable.

^b^ Measured using the Work Limitations Questionnaire (range, 0%-25%).

^c^ Measured using the Patient Health Questionnaire 9-item symptom severity score (range, 0-27).

^d^ Measured as the mean percentage of total hours missed from work in the past 2 weeks owing to health conditions or medical care divided by the number of hours usually spent working (range, 0-100).

Regarding our second hypothesis, which was tested in mixed models (Table 4; eTable 2 in Supplement 2), we found that the IC plus BWAW group advantage in at-work productivity loss at T_1_ did not erode at T_2_ (mean difference, −0.5; 95% CI, −1.9 to 0.9; *P* = .46). There was also no significant loss in the initial improvement in depression severity (mean difference, 0.6; 95% CI, −0.9 to 2.1; *P* = .44). Using mixed models, the sensitivity tests of time by group interactions (eTable 3 in Supplement 2) also supported ANCOVA results.

**Table 4.** Mixed Model Estimates for Analyses of Outcome Changes for IC-Only vs IC Plus BWAW Groups

Outcome	Estimate				Mean Difference of Changes (95% CI)	*P* Value^a^
	IC Only		IC + BWAW			
	T_1_ (n = 96)	T_2_ (n = 97)	T_1_ (n = 115)	T_2_ (n = 111)		
At-work productivity loss, %^b^	11.9	11.3	10.2	9.2	−0.5 (−1.9 to 0.9)	.46
Depression symptom severity score^c^	12.6	12.0	10.7	10.7	0.6 (−0.9 to 2.1)	.44
Productivity loss due to absence, %^d^	18.4	19.4	13.6	12.6	−2.0 (−9.0 to 5.1)	.58
Had leave of absence, %	10.4	11.4	8.8	11.4	1.5 (−8.5 to 11.5)	.76
Filed disability claim, %	5.0	7.3	3.8	6.7	0.6 (−7.0 to 8.3)	.87
Experienced job loss, %	3.0	9.3	5.1	5.4	6.0 (−0.5 to 12.5)	.07
Work hr/wk	36.9	39.9	42.8	41.1	−4.8 (−10.0 to 0.5)	.07

Abbreviations: BWAW, Be Well at Work; IC, integrated care; T_1_, 4-month follow-up; T_2_, 8-month follow-up.

^a^ Estimated based on mixed models.

^b^ Measured using the Work Limitations Questionnaire (range, 0-25).

^c^ Measured using the Patient Health Questionnaire 9-item symptom severity score (range, 0-27).

^d^ Measured as the mean percentage of total hours missed from work in the past 2 weeks owing to health conditions or medical care divided by the number of hours usually spent working (range, 1-100).

Regarding benefits, at-work productivity loss was statistically significant with a point estimate of 2.0% (95% CI, 0.8% to 3.1%) combining effects from both follow-ups ($900 marginal benefit over a 1-year period). Similarly, the point estimate for absence-related productivity loss was approximately 2.0% (95% CI, 3.6% to 7.5%) ($900 marginal benefit). The cost per BWAW participant was $690.98. Medical costs were not included because they were similar in both groups. On this basis, the total benefit is $1800, implying an ROI of 160%. Excluding gains in productivity due to absence benefits, the ROI was 30%. If the effect lasted only until the second follow-up, benefits would be smaller and the ROI would be lower, at 95%, and after excluding gains in productivity due to absence benefits, ROI would be −2%. If benefits lasted for 2 years, the total ROI would be 421%, and the ROI excluding gains in productivity due to absence benefits would be 160%.

## Discussion

This randomized clinical trial tested the benefits of adding a work-focused intervention to VHA IC for patients with depression and found that this relatively low-cost, work-focused intervention was effective and that the outcomes obtained immediately after the program ended were present 4 to 5 months later. As found in prior BWAW studies,^10,11^ the ROI was positive, partly reflecting the relatively low cost of BWAW and the high economic burden of depression.

The VHA recognizes the importance of employment to veterans’ mental health and quality of life, but it has not supplemented its integrated behavioral health interventions with work-focused services aimed at people currently employed.^3^ Currently, the Department of Veterans Affairs offers vocational supports, but these primarily serve veterans with disabilities who may need accommodations or retraining.^31^

In prior BWAW clinical trials in which participants were recruited from worksites,^10,11^ the BWAW program outperformed usual depression care, which meant any care the person elected to use. In a 2015 randomized clinical trial,^10^ BWAW was found to improve at-work productivity by 50%, productivity loss due to absences by 50%, and depression symptom severity by 50%. We found that improvements in at-work productivity, at 17%, and depression symptoms severity, at 26%, in IC plus BWAW were lower than these prior studies, which may reflect the greater health and social needs of veterans compared with other populations and the higher baseline quality of care provided in VHA IC compared with usual care. Furthermore, we found that the IC group also reduced their weekly work hours to a greater degree than the IC plus BWAW group, although they regained these hours by T_2_ so that the effect was not statistically significant. The effect on absences was also not as pronounced and not statistically significant. This may reflect the high degree of heterogeneity in workplace absence policies across the different employers, as controlling for occupation, hourly vs salaried status, unionization, paid sick days, or job tenure did not change the result. Rates of job loss and claims were similarly low. The study may have been underpowered to analyze these outcomes.

### Limitations and Strengths

While the general conculsion of this study is that IC plus BWAW is superior to IC alone, this study has some limitations. This study included only 1 VHA medical center, and many eligible veterans did not participate, limiting generalizability. The sample size may have limited our power to detect some differences, and we relied on self-reported measures of outcomes, which may contribute response bias. Additionally, we could not determine the effect of the booster session, which was not highly attended. In future studies, we may wish to compare the cost vs benefits of telephonic BWAW to in-person or video versions.

This study also had some strengths, including the study design, clinical assessment of mental health, and objective measurement of VHA service use. Our intervention was manualized and had a strong emphasis on occupational functioning as the clinical goal. Additionally, we used extensively validated self-reported measures of depression and occupational outcomes, interviewer blinding, systematic efforts to follow-up with enrollees, and care was delivered in a realistic setting.

## Conclusions

These findings suggest that adding a telephonic, work-focused intervention to IC for depression is more effective than IC alone. Facilitating reentry and reintegration into civilian life are high-priority goals within the Department of Veterans Affairs, and many employers want to hire veterans and be assured that they will function effectively in the workplace. Depression and difficulty functioning in occupational roles and settings pose significant barriers to achieving success. Building telephonic work-focused care into IC offers a holistic, accessible, and economical solution. As the VHA invests in telemedicine to address access barriers, BWAW’s telephone-based counseling provides a further opportunity to make higher-quality care more accessible to veterans.

## Notes

Supplement 1. **Trial Protocol.**

Supplement 2. **eAppendix.** Deviations From the Original Protocol **eTable 1.** Comparison of Baseline Characteristics of Study Participants With Study Drop-outs Within Integrated Care–Only Group and IC Plus Be Well at Work Group **eTable 2.** Comparison of Baseline Characteristics of Integrated Care Plus Be Well at Work Treatment Participants vs Treatment Drop-outs **eTable 3.** Model Estimates Without Baseline Data for Mixed-Model Analyses of Outcome Changes for Integrated Care–Only Group vs Integrated Care Plus Be Well at Work Group **eTable 4.** Analysis of Covariance for Last Value Carry Forward Analysis

Supplement 3. **Data Sharing Statement.**
