# Supplementary material for: Effect of Adding a Work-Focused Intervention to Integrated Care for Depression in the Veterans Health Administration: A Randomized Clinical Trial
Source: JAMA Netw Open. 2020 Feb 28;3(2):e200075. doi: 10.1001/jamanetworkopen.2020.0075 (PMC7049076; doi:10.1001/jamanetworkopen.2020.0075)
Supplement: Supplement 1. — Trial Protocol. [file jamanetwopen-3-e200075-s001.pdf]

A. Protocol Title

1. **Full Protocol Title: Reducing the Burden of Depression on Employment: Improving Function and Work**

2. **Date of Protocol Summary and Version#: Date 1/7/2014; Version# 2**

B. **Principal Investigator's Full Name and Degree: David W. Oslin, MD**

C. Co-Investigator's Full Name and Degree: N/A

D. Financial Sponsor (Provide the name of the agency, organization, company or person providing funds for the research study.) **Dept. of Veterans Affairs, Rehabilitation Research** & **Development**

E. **Grant** (Provide the name of individual who holds the grant and the grant number, if applicable.) **David Oslin 101 RX001132-01A1**

F. **Protocol Number** (Provide the financial sponsor's protocol number, if applicable.)

G. Institution(s) responsible for the project:

1. For single-site studies - PVAMC is the only institution involved. Yes No D
2. For multi-center studies.
   - 2.1. PVAMC is the Coordinating Center in which the PI is the lead investigator. Yes C No D NIA ☐
   - 2.2. Provide the name of the Coordinating Center. Yes D No D N/A D
   - 2.3. List the name of the other sites involved.
   - 2.4. Provide the FWA numbers for each of the other sites involved.
3.

<u>THE FOLLOWING INFORMATION MUST BE PVAMC-SPECIFIC. THAT IS, SPECIFIC TO WHAT WILL BE DONE WITH PVAMC-RECRUITED VETERANS.</u>

H. **Background and Significance:** (Describe succinctly and clearly the past findings which justify the plan for this project. A summary of the relevant literature in the area of interest and reports of previous studies should be included.)

Background

There is increasing concern about high unemployment and underemployment among the men and women who have served this country and the threat that the absence of gainful employment poses for the health, quality of life and economic security of veterans and their families.(13) The VA, the White House, and other public and private sector institutions l1ave **responded to this urgent crisis by committing time, energy and resources to help veterans and their spouses find adequate jobs.(14) Yet, while unemployment and underemployment are significant problems for far too many veterans, another group of veterans faces significant employment problems but receives little assistance. This is the large group of veterans who are employed but, because of their depression, are encountering formidable obstacles 1o remaining engaged and productive in their work. Existing mental health and vocational services do not address this issue directly. The proposed study will test a new mental**

health/vocational intervention, the Veterans Work and Health Initiative (V-WHI), aimed at reducing the harmful impact of depression on veterans who are already in the labor market.

Depression is a highly prevalent, chronic and recurrent condition that frequently interferes with working. In the United States (US), depression is estimated to cost billions of dollars annually in lost work productivity principally as a result of job loss, premature retirement, work disability, at-work performance limitations (known as "presenteeism") and time lost from work (absenteeism).(6;7;15) The large cost of lost productivity is related both to the prevalence of depression in the working-age population (6.4% of employed US workers)(7) and the specific manner in which it interferes with working. Since Wells and colleagues(16) first reported on the disabling impact of depression in the 1980's, evidence of its human and economic burdens has continued to accumulate.(17-20)

A large study conducted by the Tufts research team screened 14,000 primary care patients, enrolling a sample of employed adults-286 of whom had depression and another 196 depression-free healthy controls. Subjects were observed for 18 months.(21;22) Though equivalent demographically and occupationally at baseline, depressed workers were found to be limited in their ability to perform basic job tasks at levels that were four times worsu than the control groups' and they had triple the mean number absences. In addition, within six months after enrolling in the study, 15% of the depression group became unemployed; five times higher than the control group's job loss rate.(23) Moreover, the impact of depre ;sion on employment did not improve significantly over time.

The picture is bleaker for our newest veterans. In a recent study of employed OIF/OEF veterans seen in the Philadelphia **VAMC's** Behavioral Health Lab (BHL) program, multiple dimensions of job performance were impaired by depression. Work limitations were responsible for average at-work productivity losses of between 9 and 10%; equivalent to the losses seen in civilian employed populations with moderate to severe major depressiv1 disorder (MDD).(3)

<u>Service Delivery Options.</u> To our knowledge, there are no programs tailored specifically to employed veterans with depression who are having trouble at work. Various services exist that address facets of the problem, but care is fragmented and spread among a range of different programs, providers and sectors.

For example, within the Veterans Health Administration (VHA), physicians (psychiatrists and primary care providers) deliver the majority of the care that adults with depression receive. While there have been important gains such as improved access to higher quality diagnosis and treatment, especially in PACT or primary care settings, many physicians (already pressed for time) tend to focus on their patients' symptoms and treatment, not on their work problems. Ultimately, many physicians become involved in their patients' wc,rk problems around the time a disability claim is involved-which is sometimes too late to prevent it.

The VHA also has the largest and most comprehensive behavioral health services of any healthcare system. Behavioral health services are extensive but separate and include a range of acute inpatient, outpatient, emergency and consultation services, services and support for homeless veterans and intensive case management for veterans with severe mental illness (SMI). Included under behavioral health is the capacity for outpatient psychotherapy, but few psychotherapists are trained in addressing employment issues. Also within the behavioral health programs are comprehensive work training and supported employment services (e.g., for veterans with **SMI).** However, these do not target veterans who are gainfully employed. Vocational rehabilitation is available to veterans with a service-connected disability under the VetSuccess program (http://www.vba.va.gov/bln/vre/). Conceivably this program could help the portion of veterans with depression who have qualified for VA disability benefits and still work. Additionally, an array of job training, placement and employment support service:; are available to veterans (and in some cases their spouses) through the White House Joining Forces Initiative (25) (which links to the federal OneStop Employment programs), the

Wounded Warriors program and others. These programs are aimed at reducing the high unemployment rate(26) and underemployment among veterans and their families.

Finally, some adults with depression qualify for non-VA programs such as those offered in the workplace and/or the community. Many workers have access to Employee Assistance Programs (EAPs) through their employers, but seldom use them for depression assistance.(27) Community-based job training programs also could provide an option for some veterans. The Ticket to Work program is one example of an intervention to assif,t persons with disabilities with job preparation and job placement. Historically, this pro! ram rarely has been involved with helping people who are already employed. State and federal vocational rehabilitation programs also provide employment support to persons with disabilities but their services are available mainly to adults with the most severe healtl1 problems, most of whom have little or no work experience. Insurance programs, such as the US Social Security disability programs and private disability carriers, provide income replacement. Finally, The Americans with Disabilities Act(28), while potentially helping with job accommodations, has generally helped more with hiring than retention.

Taken as a whole, the V-WHI has the potential to fill a void in existing services while offering a paradigm shift for traditional mental health services from its historical focus on symptom-based pathology to a new emphasis on recovery, rehabilitation and functioning. This shift is consistent with the emerging policies from the Office of Mental Health Services and veterans' preferences. If successful, the V-WHI could be offered both to veterans i 1 VHA care and the large group of veterans who are eligible for services but choose to access care outside of the VA system.

Significance of the Research

Employed veterans with depression are vulnerable to a wide range of employment problems but a gap in their care still exists. Typically, health and work problems are addressed in separate silos and even systems. And, many interventions are geared for populations with work problems that are less intense (depressed with no work problems) or more intense (veterans with **SMI).**

Moreover, research has been surprisingly limited in its conceptualization of the mechanisms linking depression to poor work outcomes. Almost completely missing is a discussion of functional performance. Over a decade of research, the Tufts team has established that depression is related to a range of functional difficulties and these lead to worse employment and economic outcomes.

New care models that integrate psychological and work-focused interventions to achieve functional improvements may offer a solution. This study will test a new multi-modal cue program that is aimed at filling the gap. It will test whether the V-WHI will be effective in helping veterans to function effectively and productively at work. It is the first program to integrate mental health and vocational care for working veterans with depression who are already employed. If the V-WHI is effective, the proposed research will make a significant contribution to the science of functional improvement and work disability prevention. Eventually, this program could have a major impact on the health, quality of life and economic security of veterans and their families and save the VA and the nation millions of dollar::; in lost productivity.

Preliminary Studies

<u>Preliminary Research Related to V-WHI Program Development.</u> As discussed above, despite the secondary work benefits of high-quality treatment, research has shown that it is not enough to overcome the significant work disruption that depression often causes. Taking

cues from the care of problems such as musculoskeletal pain, in which return to effective functioning is regarded as an important goal, the Tufts research team concluded that employees with depression may also benefit from work-focused care addressing the medical, psychosocial and vocational dimensions of functioning. Prior research by the Tufts team and others has suggested several strategies.(29) For instance, studies of collaborative care for depression(30-35) have shown that patient clinical outcomes are better when care is organized to support provider-patient teams with care manager involvement.(33;36) Similarly, occupational medicine and disability research has shown that work-injury or disability claimants return to work more quickly when medical providers, workplace managers, and the employee collaborate.(29;37) These results imply that coordinated care involving mental health care providers, the employee, and other professionals familiar with the work situation may bridge the gap between the medical and work domains. In addition, an extensive literature demonstrates the effectiveness of cognitive-behavioral therapy (CST) in improving mental health and functioning. CST is also flexible and can be provided as a brief intervention through in-person, telephonic,(38-40) or electronic modalities.(41-45) Finally, studies of supported employment for severely and chronically adults with mental illness and oth1 r persons with disabilities offer insights into the importance of achieving a "fit" between the person and the work environment. Methods of enhancing this fit have included job coaching, providing social supports, and/or instituting modifications at work (e.g., flexible scheduling).(9;10)

V-WHI Work Coaching and Modification Component. The aim is to identify specific job performance difficulties related to depression and guide the veteran towards changing modifiable aspects of his or her work methods and/or work conditions. Conceptually, it draws upon principles articulated in research on return to work programs for work-injured employees and work disability claimants,(46) employment support for severely and chronically mentally ill populations(9;10;47;48) and disability theory(49) which consider social role performance to result from a complex person and environmental interaction.(10;50) In this WHI component, the counselor focuses initially on obtaining an accurate assessment of the participant's job and work limitations using both the WLQ and probing techniques. The counselor then elicits information from the participant regarding individual and environmental barriers that may be interfering with effective functioning. This step is followed by effo11s to guide the participant towards initiating changes aimed at improving functioning. The recommended changes are designed to be normative and safe but do not involve formal job accommodations. The recommended approaches are behavioral and/or environmental depending on the nature of the problem and the person's preferences. For example, a veteran may be having difficulty working because he feels disorganized and/or distracted. After careful assessment of his work limitations, work conditions (demands, control, supports and environmental factors) and preferences, the V-WHI counselor may suggest strategies such as using organizing tools, asking coworkers for feedback and advice and/or periodically turning off email and telephones. If disorganization and/or distraction are related to cognitive/emotional issues such as ruminating and wandering thoughts, the counselor may suggest a CST strategy.

Work-Focused Cognitive Behavioral Therapy (CST) Component. Depression is associated with maladaptive cognitions behaviors and interpersonal functioning that may interfere with work functioning. Thus, individuals with the disorder will, conceivably, benefit from developing a new repertoire of more adaptive behavioral and cognitive strategies and c::>ping skills. The WHI incorporates CST strategies originally developed by Beck;(51-53) Lewinsohn and Antonuccio, Steinmetz and Teri;(54) and adapted by others.(38-40;42-45;55) CST involves identifying and changing negative cognitions (e.g., negative self-talk, cognitive distortions, negative patterns of attribution) and maladaptive behaviors (e.g., avoidance, procrastination) that contribute to functional limitations. Studies report that CST is an

effective, time-limited alternative to more traditional therapeutic approaches to depression treatment,(56) and that it improves patients' symptoms and functional outcomes.(18;!,7) Treatment trials of enhanced primary care have shown that CBT interventions reduce social role disability.(43;58-61) CBT has also been studied within employee populations showing moderate positive effects on mental health, quality of working life, and absenteeism.(24;62-65) Recent studies using telephone-based CBT strategies have demonstrated efficacy.(66-68) These strategies are educational, goal-oriented, and time-limited collaborative endeavors that attempt to help individuals learn skills that enable more adaptive coping and functioning. The V-WHI is based on similar CBT approaches and is supported by a manual, named Creating a Balance.(69) Prior studies using telephonic CBT and Creating a Balance have demonstrated improvements in depressive symptomatology. The V-WHI has adapted Creating a Balance to emphasize the program's work focus.

<u>WHI Tests.</u> Two studies of the WHI have been completed to date and a third national study will be completed in 2013. The WHI studies were sponsored by the CDC (R01 DP000101), NIMH (R34 MH072735) and the NIA (R01 AG 033125-01A1), respectively.

The first WHI pilot test was conducted in a national aeronautics manufacturing firm. It used a quasi-experimental, pretest-posttest design with an external comparator. In that initial study, employees in the WHI program had statistically significant improvements in at-work performance and productivity based on the WLQ (described below).(8)

The second study was an RCT comparing the WHI to usual care for depression conducted in Maine State Government; Maine's largest employer. The RCT enrolled 59 employees in the WHI group and 27 non-depressed controls. Employees with other major psychiatric or physical co-morbidities were excluded. The groups were equivalent at baseline demographically, clinically and occupationally. As in the proposed study, the Maine study's primary endpoints were the WLQ at-work performance and productivity scores and health-related work absences and productivity loss based on the WLQ Time Loss Module. PHGI-9 depression severity was also assessed. The results were impressive. Although there were no baseline differences between the WHI treatment group and the usual care group (p   0.05), by follow-up the WHI treatment group had significantly better scores on every outcome and differences in the longitudinal changes were all statistically significant (p=0.027 to 0.000). The new program was superior to usual care and resulted in an estimated annualized productivity cost savings of $6000 per participant.

The third and ongoing study of the WHI is an RCT testing its effectiveness and economic impact for middle-aged and older workers with depression. The study includes 23 employers and organizations (e.g., AARP). WHI care is provided by OptumHealth EAP counselors, under the supervision of the Tufts team. This complex field trial will end enrollment in 2012. It has enrolled over 410 employed adults with depression. As seen in the two prior trials, the burden of depression is large. The frequency of work limitations is indicated by the WLQ scale scores.

Each WLQ score indicates the mean percentage of time in the 2 weeks prior to baseline that health problems limited ability to perform each category of work tasks: time management, physical tasks, mental and interpersonal tasks and output tasks (related 10 quality, quantity and timeliness of work). For example, among employees with mild depression symptom severity, work limitation scores range from being limited 19% of the time to 28% of the time. For those with moderate symptom severity, the range is 20% of the time to 42% of the time. For employees with severe symptoms, limitations range in frequency from 32% of the time to 61% of the time. The associated at-work productivity loss was between 7 to 14% on average in the three respective severity groups. Mean absences due to health or medical care in the prior two weeks ranged from just under 1 day to 2.4 days. Missed days translate into productivity losses between 7% and 22%. The burden of depression is also reflected by diagnosis. Functioning is poorest in the double depression group. Finally, the

number of co-morbid conditions, which in this study are primarily physical, was important only for performance of physical job tasks.

I. Purpose of the Project: (Clearly provide the purpose of this research project.) This study's goal is to enable employed veterans with a depression disorder to remain functionally independent and participate fully in the labor market. Its Aim 1 objective is to test the effectiveness of an innovative telephonic counseling program for employed veterans with depression, thE! Veterans Work and Health Initiative (V-WHI), which uses both psychological and voca1ional interventions. The Aim 2 objective, which is contingent on the effectiveness of the V-WHI, is to quantify its return on investment (ROI).

J. Describe the Research Questions or Hypotheses (that is, what questions are you trying to address by conducting the research.) The effectiveness of the V-WHI approach will be tested in the context of a randomized controlled trial (RCT). The following hypothesis will be tested: veterans receiving V-WHI will have better work outcomes at time 1 (T1) than the usual care group (BHL only) as measured by at-work productivity loss known as presenteeism. A sub-hypothesis for Aim 1 is that the V-WHI effect at T1 will be sustained at least until the final follow-up (T2). Secondary T1 outcome measures expected to improve include absenteeism, depression symptoms and health-related quality of life (HRQOL). Hypothesis 2 is that the return on investment (ROI in the V-WHI at time T1 will be positive, exceeding 1). Analysis of ROI will be contingent upon confirmation of Hypothesis 1. ROI will be estimated based on the marginal group difference in productivity (presenteeism and absenteeism combined) relative to the marginal V-WHI program costs.

K. Primary Outcome Variable(s): (Define the primary outcome variable(s) used to support the study objectives (e.g. if the objective is to show that treatment A is superior to treatment B in the treatment of subjects with essential hypertension, the primary outcome variable is blood pressure measurement.) The main variables for outcomes analysis quantify health-related decrements in work performance and productivity (presenteeism). In addition, we will assess work loss and work retention based on employment status and other work outcomes such as weekly hours. To help us interpret the scores, we have extensive comparative data from clinic and population-based samples for the Work Limitations Questionnaire (WLQ), the WLQ Time Loss Absence Module,(11;12) and other employment status items (e.g., job loss).

L. Secondary Outcome Variable(s): (Define the secondary outcome variables. Such measured variables should also include the timing of measurement.) The study will also measure secondary outcomes related to work including work absences related to health and/or treatment, depressive symptoms and health-related quality of life (HRQOL). Additional study endpoints will include loss of employment vs. retention of employment, new disability c:laims and change in job hours. While relevant, they occur infrequently. Depression symptom severity is measured with the PHQ-9. The HRQOL is measured with the VR-12 which generates a physical component score and a mental component score.

M. Study Design and Methods:

1. Is this a clinical trial? [g]YES ONO
   1.1. If yes, what type? Check all that apply. N/A (no medications used)
   0 Phase I O Phase II O Phase Ill O Phase IV
   1.2. If yes, this study must be registered on Clinicaltrials.gov.

2. Design
   2.1. What research methods will be used? Check all that a I .

cg] Surve s/Questionnaires	cg] Interviews	D Audio Taping
D Behavioral Observations	cg] Chart Reviews	D Video Tapin

D Focus Groups	0 Randomization	0 Double-Blind
0 Control Group	D Placebo	D Withhold/Delav Treatment
D Specimen Collection	D Deception	0 Telephone Survev
D Other (Describe)		

2.2. Describe how randomization or other treatment assignment will be made. **The RCT will involve random assignment of 250 consenting subjects. For practical reasons, neither subjects nor the intervention providers will be blinded** to subject assignment. Subject assignment will be a 50/50 ratio to V-WHI care or BHL usual care (no V-WHI care). Participants are not required to engage in BHL clinical care. Both groups are permitted to use other services through their own VA or other insurance coverage or out-of-pocket expenditures. With attrition, we **expect 225 at initial follow-up (month 4 to 5) and 200 at final follow-up (month 9); attrition rates of 10% and, in total, 20%.**

The randomization procedure will be automated within full/part-time group using a block randomization technique within group, where part-time is defined as less than 30 hours per week. Each block of 4 will contain 2 V-WHI and .2 BHL, randomly ordered. The small block sizes assure an even flow of new cases to the counselors.

2.3. For retrospective research studies, provide the "look-back" period. (e.g., December 1, 1999through December 31, 2008.) **NIA**

3. Study Duration

3.1. Provide the estimated length of time to enroll all subjects and complete the stud: . **44 months will complete subject data collection; 48 months will complete analysis, reporting, and dissemination.**

3.2. Explain the expected duration of subject participation including any follow-up. **Each V-WHI subject requires 4 months of intervention. A booster session for V-WHI subjects will be provided at 8 months post-baseline. At the end of the first intervention period, which is month 4 to 5, all subjects will be invited to complete the first follow-up survey (T1**). **At month 9 (T2), all subjects will be invited to complete a final follow-up survey.**

3.3. Specify the projected date of completion of the proposed study. **11/30/2017**

4. Drug Information (If not applicable state, "Not Applicable.") **N/A**

4.1. Specify if the drug or biological agent is:

4.1.1. FDA approved

4.1.2. Used for off-label purposes

4.1.3. Not yet FDA approved.

4.2. Include the FDA Investigational New Drug (IND) number for all non-FDA approved and off-label drugs, biological agents or nutritional supplements. If not applicable state, "Not Applicable."

4.3. Provide all relevant information about the drug

4.4. Explain any wash-out periods, rescue medications permitted and any type of medications not permitted while enrolled in the study.

4.5. Describe blinding and un-blinding procedures.

4.6. Include the dosage, route of administration, previous use, and the safety and effi,::acy information on any drug used for research purposes.

4.7. Describe rationale for the dosage in this study.

4.8. Justify why the risks are reasonable in relation to anticipated benefits and/or knowledge.

4.9. Describe where drug preparation will be done.

4.10. All drugs for PVAMC subjects must be dispensed through the VA investigational pharmacy.
4.11. Describe where the study treatment will be administered.
4.12. Describe plan for tracking a non-compliant treatment study subject.
4.13. Summarize any pre-clinical data.
4.14. Describe the process for the storage, security, dispensing and return of an investigational drug.

5. Investigational Device (If not applicable state, "Not Applicable.") **N/A**

5.1. The Investigational Device Exemption (IDE) number must be submitted for all significant risk devices and if an IDE exists for a non-significant device.
5.2. Significant Risk or Non-significant Risk - If a device is not approved by the FDA specify whether or not the sponsor has determined this device to be a "significant risk" or "non-significant risk" as defined by the FDA.
5.3. Provide all relevant information about the device.
5.4. Describe blinding and un-blinding procedures.
5.5. Specify if device is:
  5.5.1. FDA approved
  5.5.2. Used for off-label purposes
  5.5.3. Not yet FDA approved.
5.6. Explain if the investigational device will be delivered and/or stored by the Principal Investigator or Pharmacy Services.
5.7. Describe the process for the storage, security, dispensing and return of an investigational device.
5.8. For research involving an investigational device, describe the SOP or plan for device control.
5.9. Address how the device will be stored in such a way that only research staff associated with the protocol will have access to the device.
5.10. Describe measures that will be put into place to ensure that the device will only be used in participants of this research protocol.

N. **Does this project involve international research?** DYES [8JNO

1. For further instructions refer to VHA Directive 2005-050, Requirements for Conducting VA-Approved International Research Involving Human Subjects, Human Biological Specimens, or Human Data

*2. VHA Handbook 1200.05 definition of international research - VA international research is any VA-approved research conducted at international sites (not within the United States (U.S.), its territories, or Commonwealths); any VA-approved research using either human biological specimens (identified, de-identified, or coded) or human data (identified, de-identified, o(coded) originating from international sites; or any VA-approved research sending such specimens or data out of the U.S. (see par. 56). **NOTE:** For the purposes of this Handbook, research conducted at U.S. military bases, ships, or embassies is not considered international research.*

0. Study Procedure

1. Study Procedures

1.1. Outline all study procedures - *(If necessary, include a table or flow chart, showing the schedule of the procedures and interactions. Distinguish between interventions that are experimental and carried out for research purposes vs. those that are considered standard of care. Routine procedures that are performed solely for research purposes should also be identified.)*

Recruitment for this project will be through the BHL, the PVAMC's integrated care program. Based on the routine standardized BHL clinical interview,

potentially eligible veterans will be invited by the BHL staff to be referred to the study staff to hear more about the study. Potentially eligible and interested veterans will be contacted by the study staff and invited to come in to meet with the study coordinator or research assistant to sign an informed consent document and to complete a Baseline visit. Veterans will be encouraged to complete the informed consent process in-person within two weeks (if more than two weeks, the PHQ9 will be repeated). A diagnostician will meet with the patient to determine a diagnosis of depression and/or persistent depressive disorder using the DSM-5 version of the SCIO. Moderate to severe work limitations will be confirmed by the validated **Work** Limitations Questionnaire (WLQ) Productivity Loss score of 5% or more. The impact of health problems on at-work performance and productivity (presenteeism) will be measured using the WLQ 8-item short · form, taken from the BHL clinical assessment.

Consenting and eligible veterans will be enrolled and then asked to complete study baseline (pre-treatment) questionnaires. We will request the BHL assessment data from the BHL for these Veterans. In addition to the WLQ 8-item short form, the BHL assessment includes the **MINI** International Neuropsychiatric Interview(5) modules for mania, psychosis, panic disorder, generalized anxiety disorder (GAD), and alcohol abuse/dependence; the PTSD Checklist(4); alcohol use using a 7-day time line follow-back method(71); use of Illicit Substanc,3s; the 5-item Paykel scale for suicide ideation(72); and the Veterans RAND 12 Item Health Survey (VR-12).

Eligible veterans will be randomized at the baseline visit. The veteran will be informed of his/her group assignment and instructed how to proceed. For those patients assigned to the V-WHI intervention, the study therapist will have c:.ccess to both the clinical and study baseline assessment data. The assigned counselor may view the data and begin the process of scheduling the first telephone session.

The assigned V-WHI therapists will attempt to begin treatment within ·r days after assignment. The V-WHI treatment period will last for 4 months. At month 8, the V-WHI will provide a booster session. V-WHI care is entirely telephonic.

The BHL Program - Usual Care

Usual care will consist of standard behavioral health care at the Philadelphia VAMC, as offered by standard operating procedures within the BHL program. The BHL program triages Primary Care patients and engages patients with appropriate behavioral health services based on patient symptoms and patient preference. Patients are triaged to care with a BHL provider or to specialty Mental Health (MH) care. Those referred to specialty MH are enrolled in the BHL's Referral Management program, an intervention designed to enhance engagement into MH care. Treatment with a BHL provider is brief and focused and may include pharmacotherapy support using psycho-education; symptom, adherence, and side effect monitoring; and problem focused advice or action plans. Typically, care delivered within the BHL consists of one or two 30-minute sessions per month for up to 6 months. These brief and focused sessions can either be by telephone or in-person. The therapy provided as part of BHL is built upon problem-solving therapy principles, motivational interviewing principles, and the development of action plans. Finally, the BHL clinical staff are continuously assessing patients for the need for a higher level of care and facilitating the transition to more specialized services as needed. It does not duplicate any of the V-WHI components and is assumed to be complementary to them. Patients who choose to refuse clinical behavioral health care are not excluded from participating in the study.

Description of the V-WHI

The original WHI ordinarily consists of three components: 1) medical care coordination; 2) work modification and coaching interventions; and 3) work-focused cognitive behavioral therapy (CBT) strategies. For this study, medical care coordination will be provided by the VHA's BHL program. Thus, the V-WHI will consist of WHI components 2 and 3 only. In this section, we describe the V-WHI contrasting it with the BHL program.

V-WHI will consist of both work modification and coaching interventions and work-focused cognitive behavioral therapy (CBT). The V-WHI is intended 10 address barriers to recovery and functioning that occur among employed adults with depression. Barriers may occur at the individual-level and consist of cognitive, emotional and/or behavioral issues that interfere with functioning in the work context. Barriers may also occur at the environmental level and consist of psychosocial and/or physical characteristics of the workplace, job role and community. In addition, there may be supports and resources available to the person through the workplace and/or community, which potentially can contribute to achieving a positive outcome.

The Work Coaching and Modification Component of V-WHI

Depression's work impact can be manifest in a variety of ways. For example, symptoms (e.g., difficulty concentrating) can make it difficult for a person to perform specific work tasks (e.g., filing papers). Depression can impair coping strategies and diminish a person's ability to adapt to psychosocial and/or physical job demands and job stressors (e.g., variations in deadlines). It can also lead to pervasive psychological disengagement from work. Over time, the ;e problems can significantly erode a person's work performance and productivity. If unaddressed, depression may result in further damage to the worker's career, job security and quality of life.

Treatment with antidepressants and/or psychotherapy can reduce symptoms but functional deficits often persist. Thus, the approach we use is to supplement high quality medical and/or psychiatric treatment for depression with interventions aimed at changing work behavior and/or psychosocial and/01· physical work conditions. The care process involves: 1) increasing the client's awareness of depression and its impact on his or her ability to function at work; 2) identifying strategies for improving functioning that are appropriate to the problem, the person and his or her work situation; 3) providing social support while strategies are being implemented; and 4) monitoring progress and making adjustments as necessary (including referral to other available resources).
The counselors have several tools to support their efforts. These include the PHQ-9 and WLQ, which provide detailed information about the nature and severity of the client's depression symptoms and work limitations, respectively. Both assessments are repeated every four weeks. In addition, in a web-based Tools and Tips section of the study website, the counselors are provided with many examples of strategies for improving work functioning, which can be customized to the client and the job. The strategies clients are encouraged to try are not formal job accommodations. Counselors do not contact the workplace. The approach involves providing the depressed worker with a repertoire of self-help strategies aimed at functional improvement, many of which can be used even after the program ends.

The Cognitive-Behavioral Therapy (CBT) Component of V-WHI

Depression frequently involves maladaptive cognitions and behaviors that may interfere with working and contribute to depressive symptoms. Veterans, therefore, may benefit from developing a new repertoire of more positive, adaptive coping strategies. In this V-WHI component, counselors provide psycho-education to veterans about CST concepts such as the positive impact of being active and socially connected and ways in which negative self-talk can make depression worse and impair functioning. After discussing the CST concepts and how they apply to the veteran, he or she will engage in exercises to practice more positive, adaptive behavioral and cognitive strategies in everyday life. Veterans then discuss the effectiveness and impact of these new strategies with th, ir counselors. The veteran and counselor will engage in an ongoing, collaborative process of identifying maladaptive coping strategies, practicing new ones and monitoring progress. The V-WHI uses an adapted work-focused version 0 .1the manual, Creating a Balance(69).

To obtain the V-WHI, each veteran will be assigned to a study counselor who will provide the experimental treatment by telephone for 4 months. The V-WHI study counselors will not be involved in other aspects of the veteran's care. The initial 4-month period is designed to coincide with the acute phase of depression treatment. In order to support the gains made in V-WHI treatment, a booster session will be provided at 8 months post-baseline. During the initial intervention period, the veteran will have a SO-minute phone session with his or her assigned counselor every 2 weeks (for an 8-visit total). Between visits, the veteran will be asked to perform specific activities in support of treatment goals (i.e., homework). Because both V-WHI treatment components require the veteran to do homework and depression can undermine motivation, specific techniques are used to promote adherence to the care. In the V-WHI model, the counselors use Motivational Interviewing techniques to facilitate cognitive and/or behavioral change.(70) At the end of the initial intervention period, the counselor and veteran will co-write a customized self-care plan designed to maintain and extend progress. At month 8, the counselor and veteran will meet by phone to review progress (including administering the PHQ-9 and WLQ) and make any necessary adjustments in the self-care plan.

The V-WHI may also involve referring clients to appropriate sources of care and resources within the VA. For example, in the course of providing the V-WHI interventions, the counselor may become aware of concurrent health, mental health (e.g., PTSD) and/or treatment issues (e.g., an evolving substance abuse disorder) that require separate attention. Should any indication of suicide risk arise it is addressed with appropriate clinical interventions. In such cases, the counselor may elect to notify the BHL or MH provider who can coordinate the necessary care. Finally, subjects in the V-WHI do not have to be taking an antidepressant and/or participating in psychotherapy. All subjects, regardless of group, are free to participate in any care they wish to use.

1.2. Explain if and how the follow-up of subjects will occur.

Research follow-up visits can be completed over the telephone or in person. At the end of the first intervention period, which is month 4 to 5, all subjects will be invited to complete the first follow-up survey (T1). At month 9 (T2), all subjects will be invited to complete a final follow-up survey. To encourage subjects to complete each questionnaire on time, we will offer a $25 incentive for baseline and T1 assessment points, and a $45 incentive for completing the T2 assessment point. Additionally, reminder letters are mailed out shortly before each questionnaire is due and, if the person is late in responding, reminder calls will be made by the study research assistant.

1.3. Describe where, how and who will be conducting study procedures.

The V-WHI will be provided by up to counselors who have successfully completed the training provided by the Tufts team. The baseline and follow-up surveys will be completed by a research assistant. V-WHI care is entirely telephonic. The research baseline and follow-ups can be telephonic and/or in-person.

1.4. If a survey study, specify the estimated amount of time that subjects will need to complete the questionnaires/tools. The baseline and follow-up research questionnaires will take approximately 45 minutes to complete.

1.5. If a blood draw, specify the amount of blood to be drawn in milliliters and in teaspoonfuls or tablespoonfuls and specify how often and where the blood will be drawn. N/t1

2. Data Collection (Include all questionnaires and survey tools with the submission.)

2.1. Provide

2.1.1. the mode of data collection, e.g. telephone, in-person, questionnaire, interviews, Study baseline, T1, and T2 follow-ups can take place in-person or over the telephone via questionnaires. Some data will be obtained from the electronic medical records and VINCI. Utilization of the V-WHI (visits, homework assignments completed) will be obtained from 1he V-WHI counselor information system. V-WHI care is entirely telephonic. We will also request a data set from the BHL program for consented participants.

2.1.2. the precise plan for how data is to be collected or acquired

The data analyses principally will use self-report data obtained from study questionnaires. Data from these sources will be supplemented with selected variables from the BHL clinical assessment (data reques1ed from BHL), VINCI, and VA electronic health record data. Table 1 illustrates the main study variable sources. Most of the embedded questionnaires and items have been validated and most have been used in prior and ongoing depression studies.

The main variables for outcomes analysis quantify health-relat1 d decrements in work performance and productivity. In addition, wt will assess work loss and work retention based on employment status and other work outcomes such as weekly hours. To help us interpret the scores, we have extensive comparative data from clinic and population-based samples for the WLQ, the WLQ Time Loss Absence Module,(11;12) and other employment status items (e.g., job loss).

Secondary outcome variables will include depression symptom severity and HRQOL. The former is measured with the PHQ-9 (1;2). The latter is measured with the VR-12 which generates a physical component score (PCS) and a mental component score (MCS)(73).

To assist the study in determining the degree to which measured changes in WLQ and/or PHQ-9 scores are perceptible to the veteran we will have the VR-12 PCS and MCS scores. In a treatment study in which a new type of care is being evaluated, it is useful to measure the consumers' experience of care. It is important to know whether the veterans found the care to be of a high quality, a reasonable cost (in terms of personal time and effort), effective and satisfactory. To assist us in obtaining this information, we will use a modified version of the CAHPS survey for behavioral health programs (74). One of the issues we will probe specifically is veterans' preference for different types of interventions. For instance, we can evaluate perceptions of key parts of

the V-WHI such as the CBT workbook and the homework assignments. We can also probe to find out if veterans would be interested in an employer outreach component. This information can be helpful to future versions of the program.

We will also collect a range of other variables which will facili1ate analysis of the baseline comparability of the treatment groups, the impact of any differential attrition that may occur (such as the need for adjustments in the outcomes analysis), and supply rich descriptive data about veterans with depression, their work and their care. The study will assess: veteran demographics; military history; work history; current job demands autonomy and supports at work; job earnings; physical and mental health; current and past treatment for mental health and/o, behavioral health problems; utilization of VA, workplace and community support services (e.g., EAPs and vocational training); and current and past disability claims and decisions.(75)

Finally, to support the economic analysis, utilization and cost data will be collected from questions on the study's self-report surveys, the electronic health records maintained by the VHA for each veteran, and, for the experimental treatment group, the V-WHI counselor data system. The costs of delivering the V-WHI will be determined mainly on th1 basis of counselor and clinical supervision hours, which are mostly captured by the study's counselor website.

The study's surveys will contain standard open-ended questions for capturing industry and occupation. The latter will be coded according to the Bureau of the Census six-digit occupational codes. These codes give us the ability to link data to the US Bureau of Labor Statistics' O*NET system and then to impute multiple occupational characteristics regarding job demands, worker training and educational requirem,3nts and other contextual attributes of these occupations (e.g., environmental conditions). These data will also be valuable for determining the external validity of the study results.

Table 1.

Measurement Method	TO (Baseline)	T1 (first Follow-up)	T2 (Final Follow-up)
PC-SAD	X		
Chronic Condition Checklist	X		
SCIO	X		
WLQ Full Form	X	X	X
WLQ Time Loss Module	X	X	X
VR-12	X	X	X
PHQ-9	X	X	X
Demographics	X	X	X
Employment questions	X	X	X
Utilization and Costs questions	X	X	X
Disability Status		X	X
Work Loss, Retention, Turnover, Work Hours questions		X	X
CAHPS Managed Behavioral Health Survey		X	X

2.1.3. exact location where data will be collected, 8HL Clinic, MIRECC Room 8228; research assessments (baseline, T1, T2) will be entered electronically on the Data Management Unit (DMU), a secure electronic database with access restricted to approved research staff members which will reside on a VA server at the PVAMC; clinical data will be entered on the web-based counselor information system, a secure web-based study information system, which will reside on a server at the PVAMC.

2.1.4. exact location where data entry will take place. 8HL clinic, MIRECC Room 8228; research assessments (baseline, T1, T2) will be entered electronically on the Data Management Unit (DMU], a secure electronic database with access restricted to approved research staff members which will reside on a VA server at the PVAMC; clinical data will be entered on the web-based counselor information system, a secure web-based study information system, which will reside on a server at the PVAMC (VHAPHIMIRECC1).

2.1.5. the "title" of individual(s) collecting the data and analyzing the data, e.g. principal investigator, research coordinator. Principal Investigator (PII, investigators at Tufts, Senior Research Coordinator, Coordinator, Research Assistant, Statisticians/Data Analysts, study therapists/clinicians.

2.2. Provide a time line for each aspect of the study.

Table 2. Project Timetable

rO	Tusk Name	s.•art	l in,s!;	Uurat,·vn	fY1 fY1G o· 02 oi 04 0, (.V m 01	IY'I' I'/17 cy 02 Cj o" c, 0;,, 0:, 04
				mos		
2	Ph3Sf? tL Counselor Prep.=.iration			2 mos		
J	Phaso HI HCT f 8"cruitmcrt			30 nios		
5	PhJ$C V Sec.one f'o!l(WJ•Up		6/30/)01 i'	J1 fT10		
6	::>hasc VL Oat;:1 Analysis	3/3(1!/.(J·-t		41 mo		
		l\!1/?017				I

A 48-month long study is proposed (Table 2). Phase I. Study Start up. In Phase II, Counselor Preparation, 2 months **(M4-M5)** will be devoted to providing an orientation to the V-WHI study counselors The Tufts team (with WOC status) will plan and implement the V-WHI counselor training program. Training will involve performing pre/post counselor evaluations and filing documents to award counselors who successfully complete the program with continuing education credits. Phase Ill, RCT Recruitment, will allocate 30 months (M5-M35) for identifying and enrolling eligible, consenting veterans, administering the study baseline questionnaires and providing sufficient time for study subjects to complete the study intervention. During this phase, the V-WHI counselors will participate by tele-conference in refresher training sessions and weekly supervision. Throughout this phase, veteran recruitment and study participation metrics (including drop-out) will be closely monitored with corrective actions taken as necessary. In Phase IV, 1st Follow-up, involving 31 months (M9-M40), study subjects will complete follow-up questionnaires. In Phase V, 2nd Follow-up lasting 31 months (M13-M44) will complete subject data collection.

2.3. **Phase V1, Data Analysis, will overlap with Phases 11-V and beyond (M5-M46). Phase VII, Write-Up and Reporting, will occur over 4 months (M45-48).**

Chart/Records/Data Review (retrospective and/or prospective)

2.3.1. Provide the planned or approximate number of charts/records/data to Je accessed

2.3.1.1. PVAMC **NIA**

2.3.1.2. Other site **NIA**

2.3.2. Does this protocol employ an Honest Broker? DYES NO

2.3.2.1. If yes, provide name of individual.

2.3.2.2. If no, explain who will access the charts/records.

2.3.2.3. Describe from what database charts/records/data will be accessed.

3. Future Use of Data and Re-Contact, if applicable. **NIA**

3.1. If any of the participant's data are going to be retained after the study for future research, the following information must be provided to the participant:

3.1.1. Where will the data be stored?

3.1.2. Who will have access to the data?

3.2. If the subject is going to be re-contacted in the future about participating in futur3 research, this must be specified. Describe the circumstances under which the participant would be re-contacted whether within the VA or outside the VA.

3.2.1. If subjects will receive aggregate study results at the end of the study, the informed consent document must contain this information.

4. Specimen Collection: NIA

4.1. Give the source of all specimens and whether they were collected for research, treatment or diagnosis.

4.2. State where specimens will be stored, secured and when discarded.

4.3. Explain how destruction of samples will be substantiated.

P. Genetic Testing, if applicable: NIA

1. Explain if the study is looking for an association between a genetic marker and a specific disease or condition, but at this point it is not clear if the genetic marker has predictive value.

1.1. The uncertainty regarding the predictive value of the genetic marker is such that studies in this category will not involve participant counseling.

1.2. Describe if the study is based on the premise that a link between a genetic mark:!r and a specific disease or condition is such that the marker is clinically useful in predicting the development of that specific disease or condition.

1.3. Will the subject be notified of the results and the provision for genetic counseling?

O Yes O No ON/A

1.3.1. If yes, explain further.

1.4. If biological specimens are used in this protocol, please respond to the following questions b1y checking the appropriate box:

	YES	NO	N,'A
a. Does the project involve genetic testing?	☐	☐	[ ]
b. Will specimens be kept for future, unspecified use?	☐	☐	[ ]
c. Will samples be made anonymous to maintain confidentiality? *(Instructions: Note: If there is a link, it is not anonymous. Coding is not anonymous.*	☐	☐	[ ]
d. Will specimens be destroyed after the project-specific use is completed?	☐	☐	[ ]

e. Will specimens be sold in the future?	☐	☐	☐
f. Will subjects be paid for their specimens now or in the future?	☐	☐	☐
g. Will subjects be informed of the results of the specimen testinq?	☐	☐	☐
h. Are there any implications for family members based on specimen testing results? (If yes, they may be participants.)	☐	☐	☐
i. Will subjects be informed of results obtained from their DNA?	☐	☐	[ ]

1.5. Will specimens be de-identified? ☐YES ☐NO O N/A

1.5.1. If yes, please describe the procedures to be used.

1.5.2. Include at what point in the process the specimens will be de-identified.

1.6. Describe what measures will be taken to minimize the following risks from breaches of confidentiality and privacy resulting from participating in **THIS aspect** of the res:!arch project:

1.6.1. physical

1.6.2. psychological

1.6.3. financial

1.6.4. social

1.6.5. legal harm

Q. Banking of Collected Specimens

1. **Will collected specimens be banked?** DYES ONO [g]N/A

1.1. <u>IF BANKING SPECIMENS, IT MUST BE AT AN APPROVED VA REPOSITORY.</u> ,For additional information, refer to <u>VHA Handbook 1200. 12</u>, Use of Data and Data Repositories in VHA Research - March 9, 2009.)

1.2. If yes, specify the location where specimens will be banked.

1.2.1. If the location is a non-VA site, has the mandatory approval from the Chief Officer of Research and Development (CRADO) been obtained through submission of a tissue banking application <u>(VA Form 10-0436</u> - Off-site Application for an Off-site Tissue Banking Waiver)? DYES ONO []NIA

1.2.2. If applicable, attach a copy of the VA Form 10-0436

1.3. Explain how destruction of banked samples will be substantiated.

R. Subject Recruitment (characteristics of the study population)

1. **Provide the planned or targeted enrollment at:**

1.1. PVAMC - **250**

1.2. Other sites - **N/A**

1.3. Not applicable; chart review or use of previously collected data - D

2. **Screening and/or Eligibility Requirements**

2.1. Describe and provide justification for:

2.1.1. Inclusion criteria

Veterans will be considered eligible for study participation if the following criteria are met: 18 years of age (no maximum age is specified); working for pay 15 hours per week; employed in the j,:>b for 6 months (to ensure that the veteran has sufficient familiarity with the position and organization); current major depression and/or persistent depressive disorder symptoms based on DSM-5 criteria; and current work limitations. We require a minimum of 15 hours of work in a job to ensure active participation in the labor market.

2.1.2. Exclusion criteria:

non-English speaking or reading; bipolar disorder; psychosis; and/or planning to take maternity leave at any point in the next nine months.

The study will not exclude for co-morbidities such as post-traumatic stress disorder (PTSD), other anxiety disorders, alcohol and substance abuse disorders and medical illnesses as these are frequently co-occurring disorders in this population.

2.2. List all screening and/or eligibility requirements. Veterans will be considered eligible for study participation if the following criteria are met: 18 years of age (no maximum age is specified); working for pay 15 hours per week; employed in the job for 6 months; current major depression and/or persistent depressive disorder symptoms based on DSM-5 criteria; and current work limitations. Veterans will be considered ineligible if any of these criteria are met: non-English speaking or reading; bipolar disorder; psychosis; and/or planning on taking maternity leave in the next nine months. The study will NOT exclude for co-morbidities such as post-traumatic stress disorder (PTSD), other anxiety disorders, alcohol and substance abuse disorders, and medical illnesses as these are frequently co-occurring disorders in this population. After consentin£1, a diagnostician will meet with the patient to determine a diagnosis of depression and/or persistent depressive disorder using the DSM-5 version of the SCID. Prior to randomization, each participant will have to meet all study eligibility criteria.. After consenting and prior to beginning any intervention, the participant will be invited to complete the study's baseline questionnaires (TO).

2.3. Explain any special test or evaluations potential subjects may have to undergo before they are actually determined to be eligible for the study.

Potentially eligible participants will be referred by the BHL. Based on the routine standardized BHL clinical interview, veterans will be invited by the BHL staff to have their name and contact information given to the study staff, hear more about the project. Potentially eligible Veterans will be called by the study staff and invited to come in for a baseline visit. Interested veterans will meet with the study coordinator or research assistant to sign an informed consent document. Veterans will be encouraged to complete the informed consent process in-person within two weeks (if more than two weeks, the PHQ9 will be repeated). At this time, a diagnostician will meet with the patient to determine a diagnosis of depression and/or persistent depressive disorder using the DSM-5 version of the SCIO. We will use diagnosticians from the MIRECC.

Moderate to severe work limitations will be confirmed by the validated Work Limitations Questionnaire (WLQ) Productivity Loss score of 5% or more. The impact of health problems on at-work performance and productivity (presenteeism) will be measured using the WLQ 8-item short form, which is already part of the BHL clinical assessment.

2.4. Not Applicable; subjects not recruited; chart review. D

3. If applicable, indicate what populations will be targeted for recruitment as participants. Check all that apply.

Males	
Females	
Inpatients	☐
Outpatients	
VA Employees	☐
Non-English Speaking**	☐
Veteran Family members***	☐
Non-Veterans***	☐
Other (Specify)	☐

| Not Applicable, chart review | D |

3.1. **For non-English speaking subjects - If an investigator proposes to use a participant population that does not speak or read English, a copy of the translated document, as well as the English version, needs to be forwarded to the IRB for approval. Translator certification is also required.

3.2. ***If non-veterans will be recruited for this study, explain why sufficient veterans are not available to participate in the project (VHA Handbook 1200.5, paragraph 16a]. Veteran's spouses/partners, caregivers, etc. are considered non-veterans for the purposes of this study.

3.3. ***Has approval to recruit non-veterans been received from the ACOS/R&D and Medical Center Director?

3.3.1. Not Applicable

3.3.2. D Pending *(Non-veteran forms should be used. /RB office will obtain approval from ACOSIR&D and Medical Center Director.)*

4. **Does this project target a specific race or ethnic group as participants?** DYES [g] NO

If yes, check all that apply.

**Race**		**Ethnicity**	
American Indian or Alaskan Native		Hispanic or Latino	☐
Asian		Not Hispanic or Latino	☐
Black or African American	☐	Other	☐
Native Hawaiian or other Pacific Islander	☐		
Black, not of Hispanic origin	☐		
White, not of Hispanic origin	☐		
Other	☐		

4.1. Provide justification why this/these group(s) was/were chosen. **NIA**

5. **Whatthe age range of participants.?** Check all that apply.

Children (Under 18) Reguires Waiver from CRADO {VHA Directive 2001-028 Research Involving Children)	☐
Young Adults (18-21)	
Adults (22-65)	
Seniors (Over 65)	
Over 89	
Not Applicable, chart review	☐

6. **Are there specific reasons why certain populations (i.e., age, gender or ethnic groups) are excluded as participants?** DYES ONO N/A

6.1. If yes, specify reasons.

7. **Does the project require enrollment of the followig classes of participants?**

	YES	NO
a. Employees	☐	
b. Individuals with impaired decision making capability	☐	
c. Pregnant women	☐	
d. Economically and/or educationally disadvantaged persons	☐	[gJ
e. Prisoners	☐	[gJ
f. Illiterate, limited, or no English language proficiency	☐	
g, Terminally ill patients	☐	

7.1. If applicable, what is the justification for including any of the above classes of participants in the project?

7.2. If the project requires enrolling any of the above classes of participants describe any project-specific measures or special considerations, steps, or safeguards to ensure that these individuals are adequately protected.

8. Describe the exact plan how subjects will be identified and recruited for the study.

8.1. Discuss methods, e.g., referrals from physician offices, clinics, programs, or through advertisements and brochures. **Recruitment for this project will be done through referrals from the BHL.**

8.2. If using a clinic, be specific about who will identify the potential subject and how that information will be transmitted to the research staff. **BHL conducts clinical assessments on all patients referred from primary care. At the completion of the clinical assessment, all patients potentially eligible for IRB approved and 'VIIRECC approved research will be informed of these projects and asked if they can be contacted by appropriate study personnel to discuss the particular study in accordance to that protocol's procedures.**

8.3. If snowball method will be used, discuss the process and how the first individuals will be recruited. **NIA**

8.4. Describe how information will be disseminated to subjects, e.g. handouts, brochures, flyers and advertisements *(include all recruitment materials with this submissior:).* **NIA - no recruitment materials for this project.**

9. Informed Consent

9.1. Informed Consent will not be sought. D

9.2. Written informed consent from participants (VA Form 10-1086 is attached).

9.3. Written informed consent from participants' legally authorized representative (U,R) as required by VA policy and/or applicable state laws (VA Form 10-1086 is attached). D

9.4. Request Waiver of Documentation of Informed Consent D

9.5. List the **title** of the key personnel involved in the following activities:

9.5.1. Person Obtaining Consent

9.5.1.1. Provide the title(s) of individual(s) **Principal investigator, study coordinator or research assistant**

9.5.1.2. Type of training received to perform this process **Principal Investigator has completed appropriate trainings and has many years of experience in conducting research studies and providing informed consent. The project coordinator has worked in the MIRECC for 6 years as the Senior Coordinator of the research program. She has received appropriate trainings including Good Clinical Practice, Privacy and HIPAA Focused Training, VA Privacy and Information Security Awareness and Rules of Behavior, Research Compliance. It will also be assured that the research assistant has been trained appropriately as above.**

9.5.2. **Pre-Recruitment Screening** (the use of medical records and other data bases to determine populations and individuals eligible for the study), ***NIA***

9.5.3. Recruitment Process (the process in which individuals are contacted and first introduced to the study and to the possibility of participating as subjects), **Referrals will come from the BHL. Based on the BHL standardized clinical interview, potentially eligible veterans will be asked if they would like their name to be given to the study staff by the BHL staff to participate in the study. Study staff will contact the Veteran, describe**

the study briefly, answer any questions, and invite the Veteran to come in for the informed consent process and baseline visit.

9.5.4. **Informed Consent Process** (the process by which recruited subjects are fully informed about participating in the study and then formally give their voluntary consent for participating),

Potential participants will meet with the study coordinator or research assistant for the informed consent process. Veterans will be encouraged to complete the informed consent process in-person within two weeks of referral (if more than two weeks, the PHQ9 will be repeated).

The consent form will ask the participant to list one family member/designated person who may be contacted if we have difficulty locating the participant for visits. We will only contact this person in the event that we lose contact with the participant. When contacting the family member/designated person, we will inform them that the participant provided us with their contact information. In order to confirm that we are speaking to the correct person, we will confirm their relationship to the participant. We will be only asking information about how to reach the participant and will not be disclosing any clinical information. If the contact chooses not to talk to the researchers, the participant's own study participation will not be effected in any way. Refusal to provide this information will not exclude anyone from participating in the study.

9.5.5. **Screening of Recruited Subjects** (those activities in the protocol in which a final determination of eligibility of prospective subjects is made during the early phases of the study, using laboratory data, inclusion and exclusion criteria, and other person-specific information),

After signing consent, a diagnostician will meet with the participant to determine a diagnosis of depression and/or persistent depressive disorder using the SCID based on the DSM-5. Moderate to severe work limitations will be confirmed by the validated Work Limitations Questionnaire (WLQ) Productivity Loss score of 5% or more.

9.5.6. Include the breakdown of each individual's responsibilities:

9.5.6.1. Principal Investigator, - **responsible for the overall conduct of the study, primarily in providing scientific direction for the project, coordinating activities between the PVAMC and Tufts study team, ensuring adherence to best practices and regulatory requirements, and monitoring the implementation and progress of the project.**

9.5.6.2. Co-Principal Investigator **NIA**

9.5.6.3. Research Coordinator(s) - **responsible for day-to-day project coordination including supervision of the research assistant working under the supervision of the Principal Investiqator; regulatory work, including IRB submissions and maintaining study files and regulatory files.**

9.5.6.4. Additional research staff by title,

<u>Research</u> <u>Assistant</u> - consent participants and perform baseline (TO) and follow-up assessments (T1 and T2). This will include data entry and management.

<u>Statistician(s)</u> - data quality assessment and statistical analysis; participate in manuscript and presentation development. The PVAMC statistician works directly with the Tuft's team statisticians to address all data integrity questions.

<u>Local Clinical Supervisor -</u> serve as the local supervisor to the clinical staff and be the clinical liaison between the BHL providers and V-WHI therapists.

<u>Investigators</u> of Tufts Medical Center - responsible for overseeing the Tufts team, preparing and implementing the counselor training and supervision, consulting on the study website and data collection; data analysis and preparing reports, manuscripts and presentations; serve as liaison to VA medical and psychiatric personnel.

<u>Study therapists/clinicians</u> - provide the V-WHI clinical **portions of the study; responsible for collaborating/writing notes in clinical charts**

9.6. Will informed consent be obtained from potential subjects prior to determining eligibility?
YES ☐NO ☐NIA

9.6.1. If no, provide justification and a HIPAA Waiver of Individual Authorization for Disclosure of Protected Health Information.

9.7. Define when a subject is enrolled into the study, e.g. after the subject signs the informed consent or after randomized to treatment. **After the subject signs the informed consent**

9.8. Describe:

9.8.1. The process when informed consent will be obtained and protecting patients' privacy. **The veteran will meet with the study coordinator or research assistant to sign an informed consent document and HIPAA after** being **referred from the BHL. Understanding of the study will be assessed through a multiple choice consent mini quiz. Incorrect answers will be corrected and gone over until the participant understands the correct answer.**

9.8.2. Any waiting period between informing the prospective participant and obtaining consent. **There is no waiting period. Referred patients will be contacted and encouraged to come in for the informed consent process in-person within two weeks**

9.8.3. Steps taken to minimize the possibility of coercion or undue influence. **The voluntary nature of the study is underscored in the consent form. The participant's understanding of the study will be assessed through the use of a Mini Quiz during the consent process. If individuals respond incorrectly to any of the questions, they will go over those sections of the consent again, until they understand the correct answer.**

9.9. Provide the language

9.9.1. used by those obtaining consent **English**

9.9.2 understood by the prospective participant or the legally authorized representative **English**

9.10. Provide location where informed consent will be obtained. **PVAMC, MIRECC Room 8228**

10. Waiver or Alteration of Informed Consent Requirements/Waiver of Requirement to Obtain Documentation of Informed Consent

10.1. Are you requesting a waiver or alteration of informed consent? *(Check all that apply)*

10.1.1. No

10.1.2. Yes; provide justification. 0

10.1.3. Yes; for recruitment purposes only. 0

10.1.3.1. An IRS may approve a consent procedure which **<u>does not include, or which alters,</u>** some or all of the elements of informed consent set forth in this section, or waive the requirements to obtain informed consent provided the IRS finds and documents that:

- 0 1. The research involves no more than minimal risf; to the subjects;
- 0 2. The waiver or alteration will not adversely affect the rights and welfare of the subjects;
- 0 3. The research could not practicably be carried OL. t without the waiver or alteration; and
- 0 4. Whenever appropriate, the subjects will be provided with additional pertinent information after participation
- 0 5. The research or demonstration project is to be conducted by or subject to the approval of state or local government officials and is designed to study, evaluate, or otherwise examine:
    a. Public benefit or service programs;
    b. Procedures for obtaining benefits or services under those programs;
    c. Possible changes in or alternatives to those programs or procedures; or
    d. Possible changes in methods or levels of payme1t for benefits or services under those programs.

10.2. Are you requesting a waiver to obtain documentation of informed consent?

10.2.1. No

10.2.2. Yes; provide justification. 0

10.2.2.1. An IRS may **<u>waive the requirement for the investigator</u> to <u>obtain a signed consent</u>** form for some or all subjects if it finds either:

- 0 1. That the only record linking the subject and the research would be the consent document and the principal risk would be potential harm resulting from a breach of confidenti.:1lity. Each subject will be asked whether the subject wants documentation linking the subject with the research, and the subject's wishes will govern; or
- 0 2. That the research presents no more than minimal risk of harm to subjects and involves no procedures for which written consent is normally required outside of the research context.
- *<u>NOTE:</u> <u>In cases in which the documentation requirements waived, the !RB mav require the investigator to provide subiects with a written statement regarding the research</u>.*

S. **Compensation** *<u>(The amount of compensation mav not constitute an undue inducement to participate in the research.)</u>*

1. Summarize any financial compensation that will be offered to subjects. To encourage subjects to complete each questionnaire on time, we will offer incentives for each assessment point as outlined below
2. Provide the schedule for compensation. $25 each time for completing: Baseline and T1 assessments; $45 for completing T2 assessments
   - 2.1. Per study visit or session. $25 for Baseline, $25 for T1, $45 for T2
   - 2.2. Total amount for entire participation. $95
3. Explain how compensation will be provided via cash, voucher, gift card, etc. Voucher which can be redeemed in-person at the agent cashier for cash or participants may ask the study staff to submit the voucher to the agent cashier so that a check may be mailed to tl1em, We are offering this second option as some visits may be completed over the telephone and so it may be more convenient for the patient to have the check mailed.
4. If financial compensation will be prorated, explain the process. NIA
5. Not Applicable - 0

T. Withdrawal/Early Withdrawal

1. Describe how and when a subject may withdrawal from the study. The subject may choose to discontinue the study at any time. We would ask that if they wish to withdraw their consent, that they do so in writing addressed to the Pl.

   There should be no circumstances in which participation in the study needs. to be terminated by the Principal Investigator. If a participant is hospitalized or otherwise not capable of participating in sessions, when they are able to, we will restart the sessions.

   Participants who elect to discontinue from the study for any reason will be given appropriate treatment referrals. The reason the participants are discontinued from the study and any referrals made will be documented. Participants will be informed that they will be contacted for follow-up whether or not they complete the trial but that they can refuse at any point.
2. Provide procedures for the orderly termination of participation by the participant and if any consequences would result from early withdrawal from the study. The subject may choose to discontinue the study at any time. We would ask that if they wish to withdraw their consent, that they do so in writing addressed to the Pl. No consequences to the participant will result from early withdrawal.
3. Explain if survival data is required. If so, clarify how data will be obtained. NIA
4. Not Applicable; subjects not recruited; chart review. D

U. Risk/Benefit Assessment

1. Potential Study Risks
   - 1.1. Describe and assess all of the following risks that may be associated with the research:
     - 1.1.1. Physical None
     - 1.1.2. Psychological -

       The psychological assessments are completed by experienced research staff. The psychological assessment involves discussing the participants' psychiatric symptoms and history of past psychological and psychiatric difficulties and treatments. Some participants may experience distress or discomfort when discussing these issues. High levels of distress during these assessments are uncommon and our staff members are well-trained in dealing with such occurrences. We have specific operating procedures that our staff follows for high risk or patients expressing suicidal ideation.
     - 1.1.3. Social None
     - 1.1.4. Economic None
     - 1.1.5. Monetary None
     - 1.1.6. Legal None

1.1.7. Loss of confidentiality -There is the possible risk of loss of confidentiality. However, a number of protections will be put in place to maintain the confidentiality of records and reduce risks to privacy, such as training staff on privacy. Data will be stored on a secure server and will bu restricted to appropriate staff. Procedures designed to maintain confidentiality will include formal training sessions for all study personnel in the importance of confidentiality and procedures to be followed, as well as formal mechanisms for limiting access to all information that can link data to individual participants. Dr. Oslin will be responsible for working with the project staff to ensure the integrity of adherence to patient confidentiality.

1.1.8. Assess the likelihood and seriousness of such risks. Low

1.1.9. Other None

1.2. Specify what steps will be taken to minimize these risks. Staff will be trained appropriately on privacy. Data will also be stored on a secure server and will be restricted to appropriate staff. Our MIRECC and Tuft's teams have extensive experience in conducting telephone based interventions. As such we have written guidelines for managing stress or expression of suicidal ideation while on the phone. We have the capacity to work with local police and can provide di1·ect access to the VA suicide hotline. In our experience this level of intervention is rarely needed but very important for staff to be prepared for the possibility.

1.3. If methods of research create potential risks, describe other methods, if any, that were considered and why they will not be used. NIA

1.4. If chart review, breach of confidentiality is always a concern. Specify what step:5 will be taken to minimize these risks. N/A

2. Potential Study Benefits

2.1. Assess the potential benefits to be gained by the individual subject, as well as benefits that may accrue to society in general as a result of the planned work.

All participants will be informed of the anticipated risks and benefits of this study. All subjects will be offered standard care by the BHL treatment team. Therefore, no treatment will be withheld from participants. Those assigned to the V-WHI will have the opportunity to have their standard care supplemented with the s1udy intervention.

The potential benefits of this study outweigh the potential risks for participants, given the numerous procedures just described for protecting against or minimizing potential risks. All participants accepted into the study will receive close monitoring as well as treatment in an outpatient setting.

This study is the first test of a program aimed at improving the work functioning of veterans with depression who are already employed. The V-WHI reflects. current knowledge of the variables that influence work performance and productivity secondary to depression and incorporates interventions that have been shown to be beneficial in non-veteran populations. Unlike prior studies of the WHI, this will be the first study in which the program is embedded in a healthcare delivery system and coordinated with a high quality integrated care program (the BHL program). Thus, we will be subjecting this innovative program to a rigorous test of its effectiveness. In prior studies, it has been compared to a less organized usual care alternative. Additionally, the veterans in this study are likely to be somewhat more impaired (e.g. having co-morbid PTSD) than some of the employed populations studied in the past. Thus, this study is an important

step towards developing an effective VA program, leveraging the knowledge and resources we have acquired previously.

In addition to the interventions, the V-WHI will introduce a range of technological and operational advances. It includes a state-of-the art web-based clinical care system that helps to keep counselors aligned with the protocol and to have the information they need close at hand. The system also consists of assessment tools that provide validated practical information to support functional improvement.

Work plays a central part in determining the quality of life of adults in the United States and in determining the nation's economic security. By helping a segment of the population retain its ability to work and preventing productivity loss, this study may make a contribution to the quality of life and economic security of the nation's citizenry, employers and the nation. Thus, this study should achieve significant advances in research on the science and practice of functional improvement.

2.2. If the subject does not receive any direct benefit, then it must be stated here and in the consent form. **N/A**

3. Alternate Procedures

3.1 Describe the alternatives available to the subject outside the research context. **Without taking part in this research study, subjects can participate in the BHL or mental health program at the VA for depression care.**

3.2 If none, state that the alternative is not to take part in this research study at all.

V. **Data and Safety Monitoring Board (DSMB) or Data Monitoring Committee (DMC)** <u>(All Phase Ill studies are required to have a DSMB</u>. <u>*However, the !RB has the right to require a DSMB with anv study.)*</u>

1. **Will an independent DSMB or DMC oversee the project?** DYES NO ON/A

2.1. If yes, please provide contact information for the DSMB or DMC or Coordinating C:3nter Representative and attach a copy of the charter.

Name: Phone Number:

Title: E-mail:

2. **If a DSMB or DMC will not monitor this study, who will monitor this study? Check all that apply.**

Principal Investigator

Sponsor

D VA Cooperative Studies Program

Safety monitoring committee

W. **Data Monitoring** <u>*(Monitoring plans describe how a monitor, independent of the study team regularlv inspects study records to ensure the study is adhering to the study protocol and applicable research regulations and PVAMC requirements. Monitoring plans do not necessarily require the use of an independent Data and Safety Monitoring Board (OSMB)*</u>. <u>*Such independent boards are usually reserved for high-risk phase I studies, or large, mu/ti-center phase Ill trials. Federally funded studies may require the use of an independent OSMB.)*</u>

1. **Describe the data monitoring plan.** (All protocols must have a data monitoring plan appropriate for the potential risks and the complexity of the study.)

The Philadelphia VAMC Institutional Review Board requires all investigators to report serious or unexpected adverse events that occur in the context of a clinical trial. In addition, a summary of adverse effects is required to be presented as part of the annual

review process. Serious or unexpected adverse events are to be reported to the IRB within 5 days. In addition, a summary of expected and non-serious effects is reported at the time of the annual review for the IRB. The principal investigator then comments on the implications to future participants and the need for change in the risk to benefit ratio of participating in the trial.

2. Describe how protocol deviations, adverse events, serious adverse events, breaches of confidentiality, unanticipated adverse device effect (UADE), and unanticipated or unexpected problems will be reported to the PVAMC IRB and sponsor. *(Refer to the PVAMC /RB Standard Operating Procedure (SOP) Manual for reporting guidelines.)* Protocol deviations, SAEs, breaches of confidentiality will be reported to the IRB following standard IRB procedures and within the time frames outlined on the IRB SOP. AEs will be reported at the time of continuing reviews.
   2.1.
      2.1.1. Describe the management of information obtain that might be relevant to participant protections such as:
         2.1.1.1. Unanticipated problems involving risks to subjects or others
         2.1.1.2. Interim results
         2.1.1.3. Protocol modifications Participants will be informed of any of the above that alter the risk/benefit ratio of participating in the study

3. If applicable, define the plan for subjects if research shows results such as:
   3.1. Depression The intervention may involve referring clients to appropriate sources of care and resources within the VA. For example, in the course of providing the V-WHI interventions, the counselor may become aware of concurrent heal::h, mental health (e.g., PTSD) and/or treatment issues (e.g., an evolving substance abuse disorder) that require separate attention. Should any indication of suicide risk arise it is addressed with appropriate clinical interventions. In such c.;.ses, the counselor may elect to notify the BHL of MH provider who can coordinate the necessary care. All study staff are trained on appropriate procedures if subjects show signs of depression, suicide, and/or abuse.
   3.2. Suicide see above
   3.3. Abuse see above

4. Statistical Analysis
   4.1. Include statistical power calculations and the assumptions made in making thes,3 calculations.

Power Analysis and Sample Size

In this study, we wish to power the study to obtain an effect size that represents a minimally clinically important difference on the health outcomes and an economically important difference related to work productivity impact (the primary endpoint). Currently, MCID is established for the PHQ-9(2) and thu PCS and MCS for the VR-12.

For the PHQ-9, we anticipate that the average subject will have an average baseline score of 15 on a 0-27 scale. A clinically-meaningful reduction of fi points, which is stated in the literature, would have 100% power to be detected. For PCS and MCS, a clinically-meaningful 5-point improvement would afford 99% power.

With regard to Hypothesis 2, corresponding to the study's power to detect an economically important difference, the primary criterion is whether an ROI greater than 1 can be obtained. This analysis is described in the following section.

For presenteeism, a reduction of 1.2% in productivity loss (or a relative

improvement of 1.2%) would have comparable value to the maximum expucted cost of treatment, meaning that the economic value of treatment would exceed the cost. A sample size of 100 treatment and 100 controls after subject dropout would achieve an 83% power for this outcome. To be sure of getting thes, numbers based on previous success rates, we will recruit 250 subjects prior to 50-50 randomization within full time and part time strata.

4.2. Define plans for data and statistical analysis, including key elements of the statistical plan, stopping rules and endpoints.

In this section, we describe the methods for testing hypothesis 1, several additional analyses including testing related to the secondary study outcomes and sensitivity testing related to both the primary and secondary study endpoints and statistical power calculations. Following this section, there is a description of the methods for testing hypothesis 2, which consists of the ROI analysis.

Aim1. Hypothesis 1.

The primary hypothesis of the study is that presenteeism, measured by the WLQ productivity loss score (PL), will be lower in the first (T1) follow-up for the V-WHI treatment compared with IC only. Productivity loss is computed using data from the WLQ's four work limitation scales and then applying an empirically-based algorithm to the scale scores. Details of the study on which the scoring methodology was based are included in Lerner et al, 2003.(12)

For hypothesis 1, the null hypothesis that the two groups are equal. The null hypothesis is tested by fitting the ANCOVA equation:

PL1 = b*PL0 + **g*X** + c*I(V-WHI) + error

where PLO refers to the baseline WLQ productivity loss and PL1 refers to the post-treatment WLQ productivity loss, X represents a set of covariates such as full-time or part-time stratum assignment, age, gender, baseline depressive symptom severity and, prior depression, and, g represents their regression coefficients, and I(V-WHI) indicates that the veteran is assigned the experimental treatment. The regression coefficient c will be tested by at-test at 5% significance level of c=0 vs. the alternative coo. Although we believe that c<0 (the treatment reduces presenteeism) we test against inequality because a two-sided test is conservative and conventional.

The primary analysis is "intent to treat" that is, all persons assigned to each group will be followed up to the maximum possible extent, even if they drop out of IC or V-WHI treatment, and analyzed as a part of their assignment group.

Sensitivity Testing

The first type of sensitivity testing is related to issues such as randomization errors and attrition. Initially and throughout the enrollment period, a key task will be to ensure that the planned randomization procedure is working. The usual care and experimental groups cannot significantly differ on key baseline variables. The randomization will be checked by comparing the groups on baseline demographics, including age, gender, education, race, occupational category and income, health and HRQOL variables and baseline WLQ presenteeism and absenteeism scores. Ongoing monitoring of study procedures as described in Data Management should minimize randomization errors due to study implementation. However, differences could occur by chance and, thus, we will include all of the variables that are significantly different (p<0.05) between treatment groups as covariates in the regression model.

A similar approach can be used to address differential rates of drop-out. Differential dropout in the two groups can introduce bias, similar to a failure in randomization. However, if the dropout is predictable, (e.g., if a logistic regression of who drops out shows significant demographic predictors), we can add the relevant covariates to the regression model or use propensity scores to reduce bias. If drop-out is unpredictable (e.g., it appears to be outcome-related because it is related to baseline depression or WLQ scores), we can make various assumptions about what the scores might have been and analyze the data with those assumed values using selection modeling methods.

A second type of sensitivity testing concerns the possibility that different counselors will produce different outcomes). To test for a counselor effect, we can use mixed models in which the variable "counselor" is a random effect. We note that counselors are not actually chosen randomly, but may be assigned on the basis of compatible schedules with the veterans. We will estimate the overall variations contributed by counselors from mixed effect models and adjust conclusions accordingly.

Aim 1. Secondary Analyses

In this section we discuss: 1) analysis of secondary outcomes, 2) analysis of T2 outcomes, and 3) sensitivity testing of outcomes (other than the primary outcome) These include: absenteeism (time missed and productivity loss - the ratio of time missed to time expected to work), combined productivity loss due to presenteeism and absenteeism, unemployment at follow-up, job turnover, individual WLQ scale scores, the PHQ-9 severity score, and the VR-12 PCS and MCS. These are considered secondary outcomes. The impact of the V-WHI on the secondary outcomes will be tested using ANCOVA models similar to those mentioned in the discussion of the study's Hypothesis 1 analysis.

Testing for T2 outcomes (instead of T1) will determine if the intervention effects observed at T1 are maintained. These analyses will use ANCOVA models similar to those described above and applied to all outcomes.

For the additional sensitivity analyses, the methods will be similar to those described for the hypothesis 1 study endpoint. In addition, we will conduct additional analyses to address some specific issues not addressed in relation to the primary study endpoint. For example, regarding productivity loss due to absences (time missed/time expected to work), previous studies suggest that the distribution of the data is heavily skewed to the right, i.e., more people are centered around average of 10% and a few people tend to have rather large values towards 100%. In regard to that, we will specify transformations of the absenteeism data to reduce the undue influence of long right tails. Another example pertains to self-report. Because key primary and secondary outcomes will be assessed from questionnaires, and the treatment cannot be effecth1ely blinded, we will test for self-report bias. For this purpose, we can look at job loss during the post-baseline period. Job loss is a good candidate for this test because it is not an evaluative variable, but an event report. While job losses are expected to occur relatively infrequently in the study time period, we may have enough cases by final follow-up to conduct this analysis. The strong relationship between self-reported functioning, PHQ-9, and WLQ outcomes at T1 and loss of job at T2 should be equally strong in both the treatment and control groups. If treatment subjects who lose their jobs have lower WLQ and PHQ-9 scores at T1 then wewould suspect that the reported scores at T1 are too low.

A third sensitivity issue is related to the relationship of T1 and T2 outcomes and ways they might be characterized. For these purposes, we would analyze T2-T1 outcomes to state how well the results were maintained, or analyze all

measurements jointly using mixed models where there are random effects due to subject, e.g.,

$$Plit= g*X + g1*X*T1 + g2*X*T2 + a1*T1 + a2*T2 + c1*T1* I(V\text{-}WHI) + c2''T2* I(V\text{-}WHI) + ei + eit$$

where the subscript t refers to the time, 0, 1 or 2, and T1 and T2 are indicator variables corresponding to T1 and T2.

Hypothesis 2. Cost Benefit Analysis/ROI Determination.

We will analyze the ROI of the experimental program if Hypothesis 1 is confirmed and test whether, consistent with Hypothesis 2, the ROI to achieve productivity gain is greater than 1. The general approach will be to compute the cost and benefit components of the ROI. The ROI is computed as the incremental cost minus the incremental monetized benefit divided by incremental cost (amount invested in the new program). A ratio exceeding one indicates a favorable ROI.

Our Hypothesis 2 analysis will consider only that portion of the intervention's incremental cost related to the V-WHI and will quantify incremental benefit as the monetized value of productivity improvements. We will not consider consumption of resources not directly related to depression because of the complexity and scope of that type of analysis in this population. Nor will we include subject costs and benefits. Subjects presumably engage in the intervention because the resulting additional functional effectiveness and well-being outweigh their incurred costs (e.g., the time consumed). While we could measure the costs incurred by veterans in the program, we cannot measure the corresponding functional benefits in their personal lives.

Benefits: We will compute benefits by quantifying and monetizing two components of productivity loss: presenteeism and absenteeism. For each component, we will compare the average change in the usual care and V-WHI care groups. We estimate productivity losses due to presenteeism using a validated WLQ Productivity Loss algorithm. Absenteeism is computed as hours of missed work during the time period divided by hours usually worked during that time period. Each productivity loss component will be monetized by multiplying it by the sample's median (fully loaded) wage. We use the median wage because we expect the wage distribution to be right skewed, making the mean less representative of the typical worker.

The benefit (i.e., reduced productivity loss) for the V-WHI intervention can be computed as follows. Let t-,AP be the reduction in the proportion of time absent for V-WHI compared to usual care, while t-,PP is the corresponding reduction in productivity loss due to presenteeism (i.e., the reduction in the proportion of output lost to presenteeism while working). Let H be the number of work hours during the period, while W is the median fully loaded wage rate. Then saved hours of productive output for V-WHI is H x (t-,AP+t-,PP). Multiplying this result by W yields the monetized value of the reduced productivity loss.

Costs: The intervention's incremental cost has three components: The value of time counselors spend in training is the product of their (fully loaded) wage rate and the number of hours needed to complete training; 2) The incremental value of time counselors spend counseling is the product of their fully loaded wage rate and the additional hours they spend counseling (co11pared to usual care); 3) The incremental value of time patients spend being counseled is the product of their fully loaded wage rate (the median veteran wage) and the additional hours they spend in counseling (compared to usual care).

The most straightforward cost and benefit analysis would compare the value of the benefit (i.e., the value of the reduced productivity loss) to the incremental cost of counseling. Because of the project's scale, changes in benefits will then be compared with changes in incremental costs.

Finally, in addition to testing hypothesis 2, we will consider conducting an additional exploratory analysis that will investigate the implications of including changes to the cost of BHL care. Such changes are plausible when BHL care is supplied in combination with V-WHI care but we do not have any evidence to indicate what the effect may be. This analysis will use a standardized counselor wage rate for both BHL and V-WHI care. To compute potential benefits other than those related to the productivity benefits included in the base case analysis, we will assess group differences in the number of primary care visits, the number of mental health visits (e.g., therapist and psychiatrist), and number of prescriptions by medication type (e.g., antidepressant type). Changes in service utilization could conceivably be considered a change to either costs or benefits. For the purpose of our analysis, we will consider it to be a benefit.

X. **Privacy and Confidentiality** *(Privacy refers to persons and to their interest in controlling the access of others to themselves.) (Confidentiality refers to protecting information from unauthorized disclosure or intelligible interception.)* *(Investigator should contact the Privacy Officer* *for additional details.)*

1. **Indicate the type of data that will be received by the Principal Investigator. Check all that apply.**

   1.1. D De-identified - Without any identifiers that could link the data to a specific participant. (Contact Privacy Officer for assistance. *If data is coded, it is not considered de-identified.)*

   1.2. Identified - Linked to a specific participant by identifiers sufficient to identify participants. (See HIPAA and Common Rule Criteria for list of identifiers.)

   1.3. Coded - Linked to a specific subject by a code rather than a direct identifier. If coded is checked, specify:

   1.3.1 Explain who will maintain the link or code. **research coordinator *and/or* research assistant will assign and maintain the code**

   1.3.2 Describe who will have access to the link or code. **Principal Investigator, research coordinator, research assistant**

   1.3.3 Provide exact details for how the data is coded.
   Clinical and research data will be stored electronically.
   Research: Participants are assigned a study ID **number. Study data will be stored with their ID number and will not include the patients name or other identifying information with the exception of dates.**
   Clinical: In addition, a clinical information system will also be used to collect information about the V-WHI sessions. This system will assign a separate encrypted ID number and will contain patient contact information.

2. **Does the project require the use of existing Protected Health Information (PHI)** from a **database, medical records, or research records?** YES ONO ON/A

   2.1. If yes,

   2.1.1. Specify the source of the existing PHI **electronic medical record, VINCI**

   2.1.2. Indicate the specific data elements/identifiers (e.g., name, address, phone numbers, etc.) on the below table.

   2.2. If the study uses an existing database/data warehouse,

   2.2.1. Provide a description of the database/data warehouse. **VA Informatics and Computing Infrastructure (VINCI)** - **is a major informatics initiative of the Department of Veterans Affairs (VA) that provides a secure, central**

analytic platform for performing research and supporting clinical operations activities. It is a partnership between the VA Office of Information Technology (OI&T) and the Veterans Health Administration Office of Research and Development (VHA ORD). VINCI includes a cluster of servers for securely hosting suites of databases integrated from select national VA data sources.

2.2.2. Make clear who is responsible for maintaining it. VINCI servers for data, applications and virtual sessions are physically located at the VA Austin Information Technology Center (AITC), located in Austin, Texas. This secure enclave with 105 high-performance servers and 1.5 petabytes of high-speed data storage has multiple layers of security and disaster recovery to prevent data loss. VA-credentialed research or operations staff are granted access to study-specific data along with tools for analysis and reporting in the secure, virtual working environment through a certified VHA network computer within the VA.

2.2.3. Cite any relevant Standard Operating Procedures (SOP) for the database/data warehouse.

<u>VINCI: Data Collection</u>

VA provides care to Veterans at over 1,400 points of care. At the core of virtually all care processes is a broadly scoped and extensively med electronic health record system known as the Veterans Information System Technology Architecture (VistA). VistA provides a longitudinal view for patients receiving care nationwide including diagnoses, procedures, medications, labs, physiologic measurements, and text notes and reports. VA uses 130 VistA implementations to provide electronic health record services nationwide for just over 20 million Veterans historically. The aggregate content of these 130 VistA systems includes 2.3 billion documents (e.g., Progress Notes, Discharge Summaries, Reports) accumulating at a rate of 696,000 each day; 15.2 billion lab values (+1.5 million each day), 3.4 billion orders (+845,000 each day), and 1.7 billion medication administrations and prescription fills (+390,000 each day).

Data are aggregated from individual VistA systems to the VA Corporate Data Warehouse where it is modeled and prepared for use. Data published by the VHA Decision Support System (DSS), Inpatient and Outpatient Medical SAS (MedSAS), VA Health Economics Resource Center (HERC) cost data, Vital Status and VA-CMS linked data file:; maintained by VA Information Resource Center (VIReC), CDC National Death Index VA-linked data, and several other specialty data sets ,::an be requested through VINCI. VA National Data Services and other data stewards regulate the right to use the data, but VINCI facilitates the process. When study requests are approved, project-specific data are extracted from source databases and placed in SQL tables accessible only to the research team and VINCI data managers.

<u>Storage of Study Data</u>

Study data will be kept in accordance with the Department of Veterans Affairs Record Control Schedule 10-1 (RCS 10-1). Storage and transfer of any Personally Identifiable Information (PII) or Protected Health Information (PHI) must be done in accordance with applicable VA and VHA policies and directives, state and federal regulations, and applicable statutes including the Health Insurance Portability and Accountability

ACT (HIPAA). Unless explicitly requested and approved by data stewards, all sensitive patient data must remain on VINCI project servers and only aggregate data without PII / PHI may be transferred from VINCI. Any desired change in data storage location or transfer requires amending the original data request with an updated of disposition of study data. The amendment must be approved by all data stewards before data may be transferred.

Violations of data policy or approved use of data will be subject to full penalty of law, which may include suspension of access privileges, reprimand, suspension from work, demotion, removal, and criminal and civil penalties.

Upon completion of the research project, the study principal investigator in conjunction with the VA Information Security Officer (ISO), and in accordance with VA policy, will ensure that, study data containing sensitive, confidential information will be returned to the VA, sanitized and removed from all servers, desktops, removable storage devic• s, etc.

Data Access
Only study team personnel explicitly authorized by data stewards will have access to project data. The study principal investigator has the responsibility for security of study. VINCI data managers and VA OI&T personnel not under the purview of the study principal investigator control the servers, network, processors, firewall and software in the VINCI environment, including access rights granted to study personnel.

When study personnel are no longer part of the research team, the study principal investigator will amend the data access request to terminate that person's access to all study data and notify the VA Information Security Officer of such action. No sensitive patient data may be shared with anyone who does not have a VA appointment. All study team personnel with access to sensitive patient data must stay current in their required VA information security and privacy policy trainings.

2.2.4. Provide a copy of the SOP. Attached in submission.

3. Will PHI be collected prior to obtaining informed consent? [g]YES ONO ON/A
   3.1.1. If yes, complete and provide a HIPAA Waiver of Individual Authorization for Disclosure of Protected Health Information with this submission.
4. HIPAA Identifiers - Indicate the PHI that will be collected from project participants direct y or indirectly.
   4.1. [8'J Name
   4.2. [8'J All geographic subdivisions smaller than a State, including street address, city, county, precinct, zip code, and their equivalent geocodes, except for the initial three digits of a zip code if, according to the current publicly available data from the Bureau of the Census
   4.3. [8'J All elements of dates (except year) for dates directly related to an individual, and all ages over 89 and all elements of dates (including year) indicative of such age, except that such ages and elements may be aggregated into a single category of age 98 or older.
      4.3.1. ☐ Birth Date D Date of Death
      4.3.2. D Discharge date D Admission date

4.3.3. [;gJ Appointment Dates D Other Dates (e.g. lab tests, x-rays, MRI, etc.)
4.4. [;gJ Telephone numbers
4.5. D Fax numbers
4.6. D Electronic mail addresses
4.7. [;gJ Social Security Number
4.8. D Medical record numbers
4.9. D Health plan beneficiary numbers
4.10. D Account Numbers
4.11. D Certificate/license numbers
4.12. D Vehicle identifiers and serial numbers, including license plate numbers
4.13. D Device identifiers and serial numbers
4.14. D Web universal resource locators (URLS)
4.15. D Internet protocol (IP) address numbers
4.16. D Biometric identifiers, including fingerprints, voiceprints, audio recordings
4.17. D Full-face photographic images and any comparable images
4.18. D Any other unique identifying number, characteristic, or code
4.19. D Personal and Family History
4.20. D History and Physical Examination
4.21. D Discharge Summary(ies)
4.22. 0 **X-Ray**
4.23. D Diagnostic/Laboratory tests
4.24. D Drug Abuse Information
4.25. D Alcoholism or Alcohol Use
4.26. D Billing records
4.27. D Health Summary Reports
4.28. ☐Other Records:

[;gJ Progress Notes
D Photographs, videotapes, other images
D HIV (testing or infectious disease) records
D Sickle cell anemia
D Behavioral Health notes
D Operative Reports
D Medication List
D Anatomic Pathology Report

5. Will participants be contacted from existing PHI? [;gjYES ONO ON/A
   5.1. If yes, clearly explain how participants will be contacted (NOTE: this would be the same information as listed under section R.8 identification and recruitment of subjects 1. **Study staff will be given referrals from the BHL clinical staff. They will receive name, phone number and last 4 of SSN.**

6. Provide the titles of the exact individuals who will have access to the collected data. **Principal Investigator, Research Coordinator(s), Research Assistant(s), Statisticians, Investigators, study therapists/clinicians**
   6.1. Explain why these individual will have access to this data. **Necessary for participant contact, collected data, and data analysis**

Y. **Information Security** *(Contact the Information Security Officer for additional assistance regarding confidentiality (storage/security) of research data.)*

1. Provide the precise plan how data is to be collected or acquired (repeat the same inforr1ation as listed under "Data Collection" section of this form.

   V-WHI clinical care is entirely telephonic and data will be entered into the co1.mselor information system on a secure VA server. BHL usual care is telephonic and/or in- **person. Study baseline, T1, and T2 follow-up questionnaires can take place in-pe1·son or over the telephone. Some data will be obtained from the electronic medical records and VINCI. Utilization of the V-WHI (visits, homework assignments completed) will be obtained from the V-WHI counselor information system.**

   The data analyses principally will use self-report data obtained from study questionnaires. Data from these sources will be supplemented with selected variables from the BHL clinical assessment (used for eligibility screening), VINCI, and VA electronic health record data. Study questionnaires will take approximately 45 minutes

to complete. The main variables for outcomes analysis quantify health-related decrements in work performance and productivity. In addition, we will assess work loss and work retention based on employment status and other work outcomes such as weekly hours. Secondary outcome variables will include depression symptom severity and HRQOL. The former is measured with the PHQ-9. The latter is measured with the VR-12 which generates a physical component score (PCS) and a mental component score (MCS).

2. Provide a listing of the exact research data that will be stored, including but not limited to signed, original informed consent and HIPAA authorization forms, case report forms, etc. **signed, original informed consent including the mini quiz and HIPAA authorization form, all assessments, and data collected on the V-WHI intervention visits.**

3. Indicate how project's research data (original and all copies) will be stored and provide corresponding security systems. **Any paper forms, including informed consent and HIPAA, will be stored in locked cabinets in MIRECC Room B228. Clinical and research data will be stored electronically (see below). For data analysis purposes, encrypted data stripped of all PHI information will be sent to Tufts Medical center study staff via CD.**

4. PVAMC, provide exact location where research data (original and all copies) will be stored and secured. **Paper forms will be stored in MIRECC Room B228 (informed consent, HIPAA). Electronic research data will reside on several servers; the majority of the research data (regulatory documents, enrollment database) will live on the vha phif pc mirecc server, at the following path: \\VHAPHIFPCMIRECC\Research Studies\Oslin - Work Productivity. Data stored in the Data Management Unit (DMU) will on the VHAPHIHOMELESS1 server. Data stored in the Clinical database will reside on the MIRECC 1 server (\\10.36.41.70\). VINCI servers for data, applications and virtual sessions are physically located at the VA Austin Information Technology Center (AITC), located in Austin, Texas. For data analysis purposes, a copy of encrypted data stripped of all PHI information will be sent to Tufts Medical center study staff via CD.**

5. Explain how data is to be transported or transmitted from one location to another. **Encrypted data stripped of all PHI via CD.**

5.1. Informed Consent discloses PHI transported or transmitted off-site. [Z]YES ONO ☐NIA

5.2. HIPAA Authorization discloses entities to whom PHI will be transported or transmitted. D YES [Z]NO ☐NIA

5.2.1. List all entities or individuals outside PVAMC to whom data is to be disclosed, and the justification for such disclosure and the authority. **Tufts Medical Center study staff with WOC status for data analysis**

5.3. If yes, list the exact data that will be transmitted. **Research and clinical data stripped of all PHI including questionnaire responses from TO, T1, and T2, and V-WHI intervention information**

5.4. If yes, explain how data will be protected during transmission outside of PVAMC. **Encrypted data stripped of all PHI on a CD**

5.5. Off-site, provide exact location (If off-site, attach at least one of the following)

5.5.1. Data Use/Transfer Agreement DYES [Z]NO ☐NIA

5.5.2. Off-Site Storage/Transfer of Research Data [Z]YES ONO ☐NIA

5.5.3. Memorandum of Understanding DYES [Z]NO ☐NIA

5.5.4. (Note: VA data disclosed to a <u>non-VA investigator</u> at an academic affiliate for research purposes needs to be approved by <u>the Under Secretary of Health</u> or designee.)

6. List who is to have access to the data and how they are to access it (anyone who has access to the data is responsible for its security). **Principal Investigator (PI), investigators at Tufts,**

Senior Research Coordinator, Coordinator, Research Assistant, Statisticians/Data Analysts, study therapists/clinicians.

7. Describe who is to have access and be responsible for the security of the information (e.g., the Coordinating Center, the statistician, and Pl who has ultimate responsibility). **Principal Investigator (Pl), investigators at Tufts, Senior Research Coordinator, Coordinator, Research Assistant, Statisticians/Data Analysts, study therapists/clinicians.**

8. Provide mechanisms used to account for the information. **All electronic data is stored on user-restricted secure PVAMC servers. Any paper forms, including informed consent and HIPAA, will be stored in locked cabinets in MIRECC Room 8228.**

9. Give security measures that must be in place to protect individually identifiable information if collected or used. **All electronic data is stored on user-restricted secure PVAMC servers. Any paper forms, including informed consent and HIPAA, will be stored in locked cabinets in MIRECC Room 8228. Research staff members are trained on privacy and confidentiality.**

10. How and to whom a suspected or confirmed loss of VA information is to be reported. **PVAMC IRB following the PVAMC IR8 SOP.**

11. Identify any circumstances that may warrant special safeguards to protect the rights and welfare of subjects who are likely to be vulnerable including, but not limited to, those subjects who may be susceptible to coercion or undue influence, and describe appropriate actions to provide such safeguards. **N/A**

12. Electronic PHI will be stored on the following:
    12.1. PVAMC desktop computer with password protection and/or encryption. DYES [SJNO [NIA
        12.1.1. If yes, identify where the desktop is located.
    12.2. PVAMC secure server. [SJYES ☐NO ☐NIA
        12.2.1. If yes, identify the PVAMC server. **Three servers:**
        \\VHAPHIFPCMIRECC\Research Studies\Oslin - Work Productivity;
        MIRECC 1 server (10.36.41.70);
        VHAPHIHOMELESS1 server
        12.2.2. External drive that is password protected and/or encrypted. DYES JNO D N/A
            12.2.2.1. If yes, identify the external drive.
    12.3. Off-Site server DYES [SJNO ON/A <u>(If off-site, attach at least one of the follow ng.)</u>
        12.3.1. Provide exact location and the name of the <u>off-site</u> server.
        12.3.2. Data Use/Transfer Agreement DYES ONO ON/A
        12.3.3. Off-Site Storage/Transfer of Research Data [SJYES ONO ON/A
        12.3.4. Memorandum of Understanding DYES ONO ON/A

13. Explain how data is to be transported or transmitted from one location to another. **Encrypted data stripped of all PHI on a CD to Tufts Medical Center**

14. Informed Consent discloses PHI transported or transmitted off-site. [SJYES ONO ☐WA

15. HIPAA Authorization discloses entities to whom PHI will be transported or transmitted.
    [SJYES ☐NO ☐NIA

16. List all entities or individuals outside PVAMC to whom data is to be disclosed, and the justification for such disclosure and the authority. **Tufts statisticians and investigators in order for data analysis, who will be listed on the research study form once they have obtained WOC status.**

17. Clarify what protection exists for a database. **Secure server, password and staff restricted**

17.1. Data is stored:

17.1.1. With identifiers - YES ☐NO

17.1.2. Coded - YES ☐NO

17.1.3. De-Identified - ☐YES ☐NO

17.1.4. Provide the exact list of identifiers that will be stored. **Name, addre!;s(es), SS number, DOB, dates of appointments, phone number(s)**

18. Describe the plan for protecting research data from improper use or disclosure.

18.1. The Investigator must notify the Information Security Officer, Privacy Officer, IREI, Associate Chief of Staff for Research and Research Compliance Officer within one hour of the improper use or disclosure.

19. Is there a plan to apply for a Certificate of Confidentiality? DYES NO ON/A

19.1. If yes, provide a copy of the certificate with this application or to the IRB Office as soon as received.

20. Record Retention:

20.1. The required records, including the investigator's research records, must be retained until disposition instructions are approved by the National Archives and Records Administration and are published in VHA's Records Control Schedule (RCS 10-'). VHA Handbook 1200.05 §26.h

20.2. Until a schedule for local research records is published, ALL records including identifiers must be retained." ORO/ORD Guidance on Informed Consent Form Modifications Addressing VA Record Retention Requirements (July 23, 2009)

20.3. If there are additional procedures for record retention, explain further.

Z. Qualification of the Investigators

1. Provide a description of the qualifications of each investigator/co-investigator and their specific role in the study. **David Oslin, MD will serve as Principal Investigator (Pl). Dr. Oslin is a Professor of Addiction and Geriatric Psychiatry at the Philadelphia VAMC and the University of Pennsylvania. He is the Director of the VISN 4 Mental Illness, Research, Education, and Clinical Center (MIRECC). He is currently funded by a VA CSRD IIR project entitled "A Comparison of Integrated and Sequential Treatments for PTSD and Substance Use Disorders in a Population of Returning Veterans." He will be responsible for the overall conduct of the study, primarily in providing scientific direction for ·:he project, coordinating activities between the two key sites, ensuring adherence to best practices and regulatory requirements, and monitoring the implementation and progress of the project. His effort is considered part of his MIRECC related support.**

2. If applicable, the Principal Investigator must identify a qualified clinician to be responsible for all study related healthcare decisions. **David Oslin**

3. Pl should submit a current, dated CV with each new initial review.

Reference List

(1) Kroenke K, Spitzer RL, Williams JB. The PHQ-9: validity of a brief depression severity measure. *J Gen Intern Med* 2001; 16(9):606-613.

(2) Lowe B, Unutzer J, Callahan CM, Perkins AJ, Kroenke K. Monitoring depression treatment outcomes with the patient health questionnaire-9. *Med Care* 2004; 42(12):1194-1201.

(3) Adler DA, Possemato K, Mavandadi S, Lerner D, et al. Psychiatric Status and Work Performance of Veterans of Operations Enduring Freedom and Iraqi Freedom. *Psychiatr Serv* 2011; 62(1):39-46.

(4) Blanchard E, Jones-Alexander J, Buckley T, Forneris C. Psychometric properties of the PTSD checklist (PCL). *Behav Res Ther* 1996; 34(8):669-673.

(5) Sheehan D, Lecrubier Y, Sheehan K, Amorim P, Janavs J, Weiller E et al. The Mini-International Neuropsychiatric Interview (M.I.N.I): The development and validation of a structured diagnostic psychiatric interview for DSM-IV and ICD-10. *Journal of Clinical Psychiatry* 1998; 59(Sup I 20):22-33.

(6) Stewart WF, Ricci JA, Chee E, Hahn SR, Morganstein D. Cost of lost productive work time among US workers with depression. *JAMA* 2003; 289(23):3135-3144.

(7) Kessler RC, Akiskal HS, Ames M, et al. Prevalence and effects of mood disorders on work performance in a nationally representative sample of U.S. workers. *Am J Psychiatry* 2006 163(9):1561-1568.

(8) Lerner D, Adler DA, Hermann RC, Rogers WH, Chang H, Thomas P et al. Depression and Work Performance: The Work and Health Initiative Study. In: Schultz IZ, Rogers E, editors. Work Accommodation and Retention in Mental Health. New York: Springer, 2011: 103-120.

(9) Handler J, Doel K, Henry A, Lucca A. Implementing supportive employment services in a 1·eal world setting. *Psychiatr Serv* 2003; 54:960-962.

(10) Lehman AF, Goldberg R, Dixon LB, McNary S, Postrado L, Hackman A et al. Improving employment outcomes for persons with severe mental illnesses. *Arch Gen Psychiatry* 20C2; 59(2):165-172.

(11) Lerner D, Amick BC, Ill, Rogers WH, Malspeis S, Bungay K, Cynn D. The Work Limitations Questionnaire. *Med Care* 2001; 39(1):72-85.

(12) Lerner D, Amick BC, Ill, Lee JC, Rooney T, Rogers WH, Chang H et al. Relationship of employee-reportedwork limitations to work productivity. *Med Care* 2003; 41(5):649-659.

(13) Interagency Policy Committee (IPC). Strengthening Our Military Families: Meeting America's Commitment. January 2011. http://www.defense.gov/home/features /2011/0111_initiative/Strengthening_our_Military_January_2011.pdf. 2012. Washington, D.C.

(14) United States Department of Labor. Bureau of Labor Statistics.USDL-12-0493. Employment Situation of Veterans Summary. 3-20-2012.

(15) Greenberg PE, Leong SA, Birnbaum HG, Robinson RL. The economic burden of depress on with painful symptoms. *J Clin Psychiatr* 2003; 64 Suppl 7:17-23.

(16) Wells KB, Stewart A, Hays RD, Burnam MA, Rogers W, Daniels Met al. The functioning end well-being of depressed patients. Results from the Medical Outcomes Study. *JAMA* 1989; 262,)):914-919.

(17) Druss BG, Rosenheck RA, Sledge WH. Health and disability costs of depressive illness in a major U.S. corporation. *Am J Psychiatry2000;* 157(8):1274-1278.

(18) Hirschfeld RM, Dunner DL, Keitner G, Klein ON, Koran LM, Kornstein SG et al. Does psychosocial functioning improve independent of depressive symptoms?. *Biol Psychiatry* .2002; 51(2):123-133.

(19) Lin EH, VonKorff M, Russo J, Katon W, Simon GE, Unutzer J et al. Can depression treatment in primary care reduce disability? A stepped care approach. *Arch Fam Med* 2000; 9(10):1052-1058.

(20) Simon G, Katon W, Rutter C, Von Korff M, Lin E, et al. Impact of improved depression treatment in primary care on daily functioning and disability. *Psychological Medicine* 1998; 28(3):693-701.

(21) Lerner D, Adler DA, Rogers WH, Chang H, Lapitsky L, McLaughlin T et al. Work performance of employees with depression: the impact of work stressors. *Am J Health Promot* 201O; 24(3):205-213.

(22) Adler DA, McLaughlin TJ, Rogers WH, Chang H, Lapitsky L, Lerner D. Job performance deficits due to depression. *Am J Psychiatry* 2006; 163(9):1569-1576.

(23) Lerner D, Adler DA, Chang H, Lapitsky L, Hood MY, Perissinotto C et al. Unemployment, job retention, and productivity loss among employees with depression. *Psychiatr Serv* 2004; 55(12):1371-1378.
(24) Wang PS, Simon GE, Avorn J, Azocar F, Ludman EJ, McCulloch Jet al. Telephone screening, outreach, and care management for depressed workers and impact on clinical and work productivity outcomes: a randomized controlled trial. *JAMA* 2007; 298(12):1401-1411.
(25) Joining Forces: Taking Action to Serve America's Military Families. http://www.whitehouse.gov/joiningforces/resources. 2012.
(26) United States Department of Labor. Economic News Release. Table A-5. Employment status of the civilian population 18 years and over. http://www.bls.gov/news.release/empsit.t05.htm 5-14-2012.
(27) National Business Group on Health. An Employer's Guide to Employee Assistance Programs. December 2008. http://www.businessgrouphealth.org/pdfs/FINAL%20EAP_report_2008highres.pdf.
(28) Thomas VL, Gostin LO. The Americans With Disabilities Act: Shattered Aspirations and N3w Hope. *JAMA: The Journal of the American Medical Association* 2009; 301(1):95-97.
(29) Costa-Black KM, Loisel P, Anema JR, Pransky G. Back pain and work. *Best Pract Res Clin Rheumatol* 201O; 24(2):227-240.
(30) Bodenheimer T, Wagner EH, Grumbach K. Improving primary care for patients with chronic illness. *JAMA* 2002; 288(14):1775-1779.
(31) Wagner EH. The role of patient care teams in chronic disease management. *BMJ* 2000; 320(7234):569-572.
(32) Gilbody S, Bower P, Fletcher J, Richards D, Sutton AJ. Collaborative care for depression: a cumulative meta-analysis and review of longer-term outcomes. *Arch Intern Med* 2006; 166(21):2314-2321.
(33) Unutzer J, Katon W, Callahan CM, Williams JW, Jr., et al. Collaborative care management of late-life depression in the primary care setting: a randomized controlled trial. *JAMA* 2002; 288(22): 2836-2845.
(34) Katon WJ. The Institute of Medicine "Chasm" report: implications for depression collaborative care models. *Gen Hosp Psychiatry* 2003; 25(4):222-229.
(35) Gilbody S, Whitty P, Grimshaw J, Thomas R. Educational and organizational interventions to improve the management of depression in primary care: a systematic review. *JAMA* 2003; 289(23):3145-3151.
(36) Katon W, Unutzer J, Wells K, Jones L. Collaborative depression care: history, evolution and ways to enhance dissemination and sustainability. *Gen Hosp Psychiatry* 201O; 32(5):456-464.
(37) Shaw W, Hong Q, Pransky G, Loisel P. A literature review describing the role of return-to-work coordinators in trial programs and interventions designed to prevent workplace disability. *J Occup Rehabi/2008;* 18(1):2-15.
(38) Fava GA, Rafanelli C, Grandi S, Canestrari R, Morphy MA. Six-year outcome for cognitive behavioral treatment of residual symptoms in major depression. *Am J Psychiatry* 1998; 155(10):1443-1445.
(39) Jacobson NS, Dobson KS, Truax PA, Addis ME, Koerner K, Golian JK et al. A componen: analysis of cognitive-behavioral treatment for depression. *J Consult Clin Psychol* 1996; 6L.(2):295-304.
(40) Katon W, Von Korff M, Lin E, Simon G, et al. Stepped collaborative care for primary care patients with persistent symptoms of depression: a randomized trial. *Arch Gen Psychiatry* 1999; 56(12):1109-1115.
(41) Callahan CM, Hendrie HC, Dittus RS, Brater DC, Hui SL, Tierney WM. Improving treatment of late life depression in primary care: a randomized clinical trial. *J Am Geriatr Soc* 1994; 42:8):839-846.
(42) Jarrett RB, Kraft D, Doyle J, et. al. Preventing recurrent depression using cognitive therapy with and without a continuation phase: a randomized clinical trial. *Arch Gen Psychiatry* 2001; 58(4):381-388.

(43) Ludman E, Von Korff M, Katon W, et al. The design, implementation, and acceptance of c1 primary care-based intervention to prevent depression relapse. *Int J Psychiatry Med* 2000 30(3):229-245.

(44)· Ludman E, Bush T, Lin E, Von Korff M, Simon G, Katon Wet al. Behavioral factors associ3ted with medication adherence and symptom outcomes in a primary care-based depression prevention intervention trial. *Ann Behav Med* 2001; 23(supp):S034.

(45) Paykel ES, Scott J, Teasdale JD, Johnson AL, Garland A, Moore R et al. Prevention of relapse in residual depression by cognitive therapy: a controlled trial. *Arch Gen Psychiatry* 1999; 56(9):829-835.

(46) Goetzel RZ, Guindon AM, et al. Health and productivity management: establishing key performance measures, benchmarks, and best practices. *J Occup Environ Med* 2001; 43(1):10-17.

(47) Kopelowicz A, Liberman RP. Integrating treatment with rehabilitation for persons with major mental illnesses. *Psychiatr Serv* 2003; 54(11):1491-1498.

(48) Loisel P, Durand MJ. Disability prevention: the new paradigm of management of occupational back pain. *Disease Management and Health Outcomes* 2001; 9(7):351-360.

(49) Albrecht GL, Seelman KO, Bury (eds). Handbook of Disability Studies. Thousand Oaks: Sage, 2001.

(50) Ellwood OT, Blank RM, Blasi J, Kruse D, Niskanen WA, Lynn-Dyson K. A Working Nation: Workers, Work, and Government in the New Economy. New York: Russell Sage Foundation, 2000.

(51) Beck AT. Cognitive Therapy and the Emotional Disorders. New York: International Univ. Fress, 1979.

(52) Beck AT. Cognitive therapy. A 30-year retrospective. *Am Psychol* 1991; 46(4):368-375.

(53) Beck J. Cognitive Therapy: Basics and Beyond. New York: Guildford, 1995.

(54) Lewinsohn PM, Antonuccio DA, Steinmetz J, Teri L. The coping with depression course: a psychoeducational intervention for unipolar depression. Eugene, OR: Castalia Press, 1984.

(55) Scott J, Teasdale JD, Paykel ES, Johnson AL, et al. Effects of cognitive therapy on psychological symptoms and social functioning in residual depression. *Br J Psychiatry* 2000; 177:440-4L-6.

(56) Brewin CR. Theoretical foundations of cognitive-behavior therapy for anxiety and depress on. *Annu Rev Psychol* 1996; 47(1):33-57.

(57) Keller MB, McCullough JP, Klein ON, Arnow B, Dunner DL, Gelenberg AJ et al. A comparison of nefazodone, the cognitive behavioral-analysis system of psychotherapy, and their combin3tion for the treatment of chronic depression. *N Engl J Med* 2000; 342(20):1462-1470.

(58) Katon W, Ludman E, Simon G, Lin E, Von KM, Walker E et al. The Depression Helpbook. Boulder, CO: Bull Publishing, 2002.

(59) Katon W, Rutter C, Ludman EJ, Von Korff **M,** Lin E, Simon Get al. A randomized trial of relapse prevention of depression in primary care. *Arch Gen Psychiatry* 2001; 58(3):241-247.

(60) Rost K, Nutting P, Smith J, Werner J, Duan **N.** Improving depression outcomes in community primary care practice: a randomized trial of the quEST intervention. Quality Enhancement by Strategic Teaming. *J Gen Intern Med* 2001; 16(3):143-149..

(61) Wells KB, Sherbourne C, Schoenbaum M, Duan **N,** et al. Impact of disseminating quality improvement programs for depression in managed primary care. *JAMA* 2000; 283(2):212-220.

(62) Marhold C, Linton SJ, Melin L. A cognitive-behavioral return-to-work program: effects on pain patients with a history of long-term versus short-term sick leave. *Pain* 2001; 91(1-2):155-163.

(63) Marhold C, Linton SJ, Melin L. Identification of obstacles for chronic pain patients to return to work: evaluation of a questionnaire. *J Occup Rehabil* 2002; 12(2):65-75.

(64) van der Klink JJ, Blank RW, Schene AH, van Dijk FJ. Reducing long term sickness absence by an activating intervention in adjustment disorders: a cluster randomised controlled design. *Occup Environ Med* 2003; 60(6):429-437.

(65) van der Klink JJ, Blank RW, Schene AH, van Dijk FJ. The benefits of interventions for work-related stress. *Am J Public Health* 2001; 91(2):270-276.

(66) Ludman EJ, Simon GE, et al. A randomized trial of telephone psychotherapy and pharmacotherapy for depression: continuation and durability of effects. *J Consult Clin Psycho/* 2007; 75(2):257-266.

(67) Lynch DJ, Tamburrino MB, Nagel R. Telephone Counseling for Patients with Minor Depression: Preliminary Findings in a Family Practice Setting. *The Journal of Family Practice* 1997; 44(3):293-298.

(68) Mohr DC, Hart SL, Julian L, Catledge C, Hones-Webb L, Vella Let al. Telephone-administered psychotherapy for depression. *Arch Gen Psychiatry* 2005; 62(9):1007-1014.

(69) Simon G, Ludman E, Tutty S. Creating a Balance: A Step by Step Approach to Managing Stress and Lifting Your Mood. Victoria, B.C: Trafford Publishing (copyright, Group Health Cooperative), 2006.

(70) Miller WR, Rollnick S. Motivational Interviewing: Preparing People for Change, second edition. 2 ed. New York, NY: Guilford Press, 2000.

(71) Sobel! L, Brown J, Leo G, Sobell M. The reliability of the Alcohol Timeline Followback when administered by telephone and by computer. *Drug Alcohol Depend* 1996; 42(1):49-54.

(72) Paykel ES, Myers J, Lindenthal J, Tanner J. Suicidal feelings in the general population: a prevalence study. *British Journal of Psychiatry* 1974; 124(0):460-469.

(73) Ware JE Jr, Sherbourne CD, Davies A, et al. A short-form general health survey. WD-2544-1-HJKFF. 1995. Santa Monica, CA, The Rand Corporation.

(74) U.S Department of Health and Human Services.AHRQ. CAHPS Surveys and Tools to Advance Patient-Centered Care. http://www.cahps.ahrq.gov/About-CAHPS.aspx . 2012.

(75) Karasek R, Brisson C, Kawakami N, Houtman I, Bongers P, Amick B. The Job Content Questionnaire (JCQ): an instrument for internationally comparative assessments of psychosocial job characteristics. *J Occup Health Psychol* 1998; 3(4):322-355.
