# Supplementary material for: Effect of Adding a Work-Focused Intervention to Integrated Care for Depression in the Veterans Health Administration: A Randomized Clinical Trial
Source: JAMA Netw Open. 2020 Feb 28;3(2):e200075. doi: 10.1001/jamanetworkopen.2020.0075 (PMC7049076; doi:10.1001/jamanetworkopen.2020.0075)
Supplement: Supplement 2. — eAppendix. Deviations From the Original Protocol eTable 1. Comparison of Baseline Characteristics of Study Participants With Study Drop-outs Within Integrated Care–Only Group and IC Plus Be Well at Work Group eTable 2. Comparison of Baseline Characteristics of Integrated Care Plus Be Well at Work Treatment Participants vs Treatment Drop-outs eTable 3. Model Estimates Without Baseline Data for Mixed-Model Analyses of Outcome Changes for Integrated Care–Only Group vs Integrated Care Plus Be Well at Work Group eTable 4. Analysis of Covariance for Last Value Carry Forward Analysis [file jamanetwopen-3-e200075-s002.pdf]

Supplementary Online Content

Lerner D, Adler DA, Rogers WH, Ingram E, Oslin DW. Effect of adding a work-focused intervention to integrated care for depression in the Veterans Health Administration: a randomized clinical trial. *JAMA Netw Open*. 2020;3(2):e200075. doi:10.1001/jamanetworkopen.2020.0075

eAppendix. Deviations From the Original Protocol

eTable 1. Comparison of Baseline Characteristics of Study Participants With Study Drop-outs Within Integrated Care–Only Group and IC Plus Be Well at Work Group

eTable 2. Comparison of Baseline Characteristics of Integrated Care Plus Be Well at Work Treatment Participants vs Treatment Drop-outs

eTable 3. Model Estimates Without Baseline Data for Mixed-Model Analyses of Outcome Changes for Integrated Care–Only Group vs Integrated Care Plus Be Well at Work Group

eTable 4. Analysis of Covariance for Last Value Carry Forward Analysis

This supplementary material has been provided by the authors to give readers additional information about their work.

© 2020 Lerner D et al. *JAMA Network Open.*

eAppendix. Deviations From the Original Protocol

There were some other minor deviations from the original protocol. The SF-12 Veterans Health Survey (VR-12) was going to be administered at baseline and both follow-ups, but it was only administered at baseline (to reduce respondent burden). Finally, we did not test for counselor effects in the experimental treatment group because one of two counselors had a higher caseload.

© 2020 Lerner D et al. *JAMA Network Open.*

eTable 1. Comparison of Baseline Characteristics of Study Participants With Study Drop-outs Within Integrated Care–Only Group and IC Plus Be Well at Work Group

	IC Participants	IC Drop-Outs	p	IC+BWAW Participants	IC+BWAW Drop-Outs	p (diff)
N	104	10		127	12	
Baseline						
Percentage At-Work Productivity Loss,[a] mean (SD)	12.2	13.4	.43	12.3	13.2	.53
Depression Symptom Severity,[b] mean (SD)	14.5	12.1	.12	14.4	15.0	.69
Percentage Productivity Loss Absences,[c] mean (SD)	20.6	21.3	.94	17.3	13.6	.55
Had Leave of Absence, No. (%)	14.7	10.0	.69	12.8	0.0	.19
Filed Disability Claim, No. (%)	6.7	0.0	.40	2.4	0.0	.59
Weekly Work Hours, mean (SD)	45.6	50.6	.44	43.7	46.8	.52
Age, mean (SD)	45.4	38.5	.07	46.8	43.2	.30
Male, No. (%)	83.7	90.0	.60	86.6	100.0	.18

[a]Work Limitations Questionnaire scores indicate the mean percentage of time in the prior two weeks the person was limited by health conditions. At-work productivity loss score from the Work Limitations Questionnaire (range=0-25).
[b]PHQ-9 symptom severity score (range=0-27).
[c]Productivity loss is the mean percentage of total hours missed from work in the past two weeks due to health conditions or medical care divided by the number of hours usually spent working (range=0-100).

© 2020 Lerner D et al. *JAMA Network Open.*

eTable 2. Comparison of Baseline Characteristics of Integrated Care Plus Be Well at Work Treatment Participants vs Treatment Drop-outs

	IC+BWAW Participants	IC+BWAW Drop-Outs	p (diff)
N	122	17	
Baseline			
Percentage At-Work Productivity Loss,[a] mean (SD)	12.4	12.2	.89
Depression Symptom Severity,[b] mean (SD)	14.5	14.2	.84
Percentage Productivity Loss Absences,[c] mean (SD)	17.5	12.6	.34
Had Leave of Absence, No. (%)	12.5	5.9	.43
Filed Disability Claim, No. (%)	2.5	0.0	.51
Weekly Work Hours, mean (SD)	44.5	39.9	.27
Age, mean (SD)	46.9	43.5	.26
Male No.(%)	86.9	94.1	.40

[a]Work Limitations Questionnaire scores indicate the mean percentage of time in the prior two weeks the person was limited by health conditions. At-work productivity loss score from the Work Limitations Questionnaire (range=0-25).
[b]PHQ-9 symptom severity score (range=0-27).
[c]Productivity loss is the mean percentage of total hours missed from work in the past two weeks due to health conditions or medical care divided by the number of hours usually spent working (range=0-100).

© 2020 Lerner D et al. *JAMA Network Open.*

eTable 3. Model Estimates Without Baseline Data for Mixed-Model Analyses of Outcome Changes for Integrated Care–Only Group vs Integrated Care Plus Be Well at Work Group

	IC T_1[1]	IC T_2[1]	IC Change	p	IC+ BWAW T_1[1]	IC+ BWAW T_2[1]	Change	p	Mean Diff. of Changes	p
N	89	89			99	99				
Primary Outcome										
Mean Percentage of At-Work Prod. Loss[b]	11.7	11.4	-0.4	0.40	9.8	9.1	-0.6	0.07	-0.5	0.46
Secondary Outcomes										
Mean Depression Symptom Severity[c]	12.3	12.1	-0.2	0.68	10.6	10.8	0.2	0.64	0.6	0.44
Mean Percentage Prod. Loss Absences[d]	17.3	19.2	1.9	0.54	14.1	12.4	-1.7	0.43	-1.9	0.58
Percentage Had Leave of Absence	9.5	10.7	1.2	0.77	9.4	10.4	1.0	0.76	1.5	0.76
Percentage Filed Disability Claim	6.0	8.3	2.4	0.53	2.1	5.2	3.1	0.18	0.6	0.87
Percentage Job Loss	2.9	9.3	6.4	0.02	4.9	5.3	0.4	0.88	-6.0	0.12
Mean Weekly Work Hours	37.9	40.1	2.2	0.17	42.9	40.3	-2.6	0.13	-4.8	0.07

[a]Data were obtained at baseline (T_0), four-month follow-up (T_1) and eight-month follow-up (T_2). IC+BWAW intervention was four months in duration. Follow-up values are estimated based on mixed models.
[b]At-work productivity loss score from the Work Limitations Questionnaire (range=0-25).
[c]PHQ-9 symptom severity score (range=0-27).
[d]Productivity loss is the mean percentage of total hours missed from work in the past two weeks due to health conditions or medical care divided by the number of hours usually spent working (range=0-100).

© 2020 Lerner D et al. *JAMA Network Open.*

eTable 4. Analysis of Covariance for Last Value Carry Forward Analysis

	IC T_0	IC T_1	IC Change, mean (95% CI)	IC p	IC+BWAWT_0	IC+BWAWT_1	IC+BWAW Change, Mean (95% CI)	IC+ BWAWp	Adjusted Effect (95% CI)	p
N	114	114			139	139				
Mean Percentage of At-Work Productivity Loss[a]	12.3	11.5	-0.8 (-1.7, 0.0)	.07	12.4	10.1	-2.3 (-3.1,-1.4)	<.001	-1.4 (-2.6,-0.3)	.02
Mean Depression Symptom Severity[b]	14.3	12.3	-2.0 (-3.0,-1.0)	<.001	14.4	10.9	-3.5 (-4.5,-2.5)	<.001	-1.5 (-2.8,-0.1)	.03
Mean Percentage Prod. Loss Absences[c]	20.7	19.3	-1.3 (-5.7, 3.0)	.55	16.9	13.3	-3.6 (-7.1,-0.2)	.04	-4.6 (-8.9,-0.3)	.04

[a]At-work productivity loss score from the Work Limitations Questionnaire (range=0-25).
[b]PHQ-9 symptom severity score (range=0-27).
[c] Productivity loss is the mean percentage of total hours missed from work in the past two weeks due to health conditions or medical care divided by the number of hours usually spent working (range=0-100).

© 2020 Lerner D et al. *JAMA Network Open.*
