# Supplementary material for: Effect of Adding a Work-Focused Intervention to Integrated Care for Depression in the Veterans Health Administration: A Randomized Clinical Trial
Source: JAMA Netw Open. 2020 Feb 28;3(2):e200075. doi: 10.1001/jamanetworkopen.2020.0075 (PMC7049076; doi:10.1001/jamanetworkopen.2020.0075)
Supplement: Supplement 3. — Data Sharing Statement. [file jamanetwopen-3-e200075-s003.pdf]

Data Sharing Statement

Lerner. Effect of Adding a Work-Focused Intervention to Integrated Care for Depression in the Veterans Health Administration. *JAMA Netw Open*. Published February 28, 2020. 10.1001/jamanetworkopen.2020.0075

Data
Data available: Yes
Data types: Deidentified participant data, Data dictionary
How to access data: Dave.Oslin@va.gov
When available: With publication

Supporting Documents
Document types: None

Additional Information
Who can access the data: Researchers whose proposed use of the data has been approved.
Types of analyses: Research purposes.
Mechanisms of data availability: After approval of a proposal and with a signed data access agreement as required by the VA.
Any additional restrictions: None
